# Bend family proteins mark chromatin boundaries and synergistically promote early germ cell differentiation

**DOI:** 10.1007/s13238-021-00884-1

**Published:** 2021-11-03

**Authors:** Guang Shi, Yaofu Bai, Xiya Zhang, Junfeng Su, Junjie Pang, Quanyuan He, Pengguihang Zeng, Junjun Ding, Yuanyan Xiong, Jingran Zhang, Jingwen Wang, Dan Liu, Wenbin Ma, Junjiu Huang, Zhou Songyang

**Affiliations:** 1 MOE Key Laboratory of Gene Function and Regulation, Guangzhou Key Laboratory of Healthy Aging Research, School of Life Sciences, Sun Yat-sen University, Guangzhou, 510275 China; 2 Sun Yat-sen Memorial Hospital, Sun Yat-sen University, Guangzhou, 510120 China; 3 Zhongshan School of Medicine, Sun Yat-sen University, Guangzhou, 510080 China; 4 Verna and Marrs Mclean Department of Biochemistry and Molecular Biology, Baylor College of Medicine, One Baylor Plaza, Houston, TX 77030 USA; 5 Bioland Laboratory, Guangzhou, 510320 China; 6 Center for Reproductive Medicine, The Third Affiliated Hospital of Sun Yat-sen University, Sun Yat-sen University, Guangzhou, 510630 China

**Keywords:** embryonic stem cell, self-renewal and differentiation, early development, chromatin organization, Bend5 and Bend4

## Abstract

Understanding the regulatory networks for germ cell fate specification is necessary to developing strategies for improving the efficiency of germ cell production *in vitro*. In this study, we developed a coupled screening strategy that took advantage of an arrayed bi-molecular fluorescence complementation (BiFC) platform for protein-protein interaction screens and epiblast-like cell (EpiLC)-induction assays using reporter mouse embryonic stem cells (mESCs). Investigation of candidate interaction partners of core human pluripotent factors OCT4, NANOG, KLF4 and SOX2 in EpiLC differentiation assays identified novel primordial germ cell (PGC)-inducing factors including BEN-domain (BEND/Bend) family members. Through RNA-seq, ChIP-seq, and ATAC-seq analyses, we showed that Bend5 worked together with Bend4 and helped mark chromatin boundaries to promote EpiLC induction *in vitro*. Our findings suggest that BEND/Bend proteins represent a new family of transcriptional modulators and chromatin boundary factors that participate in gene expression regulation during early germline development.

**Supplementary Information:**

The online version contains supplementary material available at 10.1007/s13238-021-00884-1.

## INTRODUCTION

Understanding the mechanisms of germ cell lineage development is central to reproductive medicine and offers valuable insight into origin-dependent imprinting as well as imprinting disorders. Despite the differences between human and mouse germ cell development, *in vitro* and *in vivo* mouse models remain indispensable due to technical and ethical constraints in studying human development. Indeed, studies in mouse have proven especially informative, revealing a spatially and temporally coordinated developmental process that progresses from primordial germ cell (PGC) specification (E6.25–7.25) to PGC migration and gonadal ridge formation (E8.0–12.5) and sex determination (after E13.5) (Saitou and Yamaji, 2010). Once arriving at the gonadal ridge, PGCs can differentiate into either oocytes or spermatozoa (Saitou and Yamaji, 2010; Saitou et al., 2012).

Epigenetic regulation (e.g., DNA demethylation, histone modifications, and chromatin remodeling) is integral to germ cell development (Kurimoto et al., 2015; von Meyenn et al., 2016). For instance, DNA methylation peaks during PGC specification and then declines during PGC migration before reaching its lowest level at E13.5 (Nakaki et al., 2013; Smith and Meissner, 2013). Throughout extensive epigenetic reprogramming occurs, including the erasure of parental imprints and reestablishment of DNA methylation in gametes (von Meyenn et al., 2016). DNA methylation erasure is an active demethylation process (Hajkova et al., 2002; Lee et al., 2002), mediated by ten-eleven translocation (Tet) proteins that catalyze the hydroxylation of 5-methylcytosine (5mC) to 5hmC (Wu and Zhang, 2017; Hill et al., 2018; Verma et al., 2018). Tet methylcytosine dioxygenase 1 and Tet methylcytosine dioxygenase 2 (*Tet1*/*Tet2)*-deficient mice have abnormal methylation at imprint loci (Zhang et al., 2010; Hackett et al., 2013; Vincent et al., 2013; Yamaguchi et al., 2013; Okashita et al., 2014).

Many factors, such as the TGFβ superfamily, are known to play crucial roles in germ cell development (de Sousa Lopes et al., 2004; Saitou and Yamaji, 2010; Senft et al., 2019). Another well-known marker for established PGCs is developmental pluripotency-associated 3 (Dppa3/Stella), which is expressed in oocytes, blastocysts, and during PGC development *in vivo* (Payer et al., 2006). Although *Stella* deficiency alone does not affect PGC fate (Bortvin et al., 2004), its overexpression can promote germline differentiation (Wongtrakoongate et al., 2013). It was shown recently that Tet protein inactivation could downregulate *Stella* expression in mouse embryonic stem cells (mESCs) (Mulholland et al., 2020), indicating a role of DNA methylation in regulating *Stella* expression, although how *Stella* is upregulated during early embryonic development for PGC commitment remains unclear. Several PR/SET domain zinc finger proteins (e.g., Prdm1/Blimp1 and Prdm14), which can function as chromatin remodeling and transcription factors, are also critical to early PGC specification. For example, Prdm1 or Prdm14 knockout (KO) mouse embryos lacked PGCs (Ohinata et al., 2005; Yamaji et al., 2008; Kurimoto et al., 2008a, b; Vincent et al., 2013). Prdm14 could also associate with Tet1 and enhance Tet1 recruitment to demethylate germ line-specific genes and imprinted loci in mESCs (Okashita et al., 2014).

In the past decade researchers have successfully recapitulated development *in vitro*, generating mouse PGCs that were capable of developing into gametes and viable animals (Hayashi et al., 2012; Nakaki et al., 2013; Hikabe et al., 2016). For example, through a two-step culturing scheme, pluripotent mESCs can be converted to epiblast-like cells (EpiLCs) and then PGC-like cells (PGCLCs) (Hayashi et al., 2011, 2012). The efficiency of EpiLC/PGCLC derivation from mESCs remains low, even with the overexpression of PGC specification master regulators such as *Prdm1*, *Stella*, *Prdm14*, and transcription factor AP-2 gamma (*Tfap2c*) or pluripotency factors such as Nanog homeobox (*Nanog*) (Nakaki et al., 2013; Murakami et al., 2016). Pluripotency transcription factors are key to maintaining pluripotency and sufficient to reprogram differentiated cells into pluripotent stem cells (Loh et al., 2006; Takahashi and Yamanaka, 2006; van den Berg et al., 2010). In addition to mESCs and primed epiblast cells, they are also expressed in PGCs and critical to germ cell lineage determination (Kehler et al., 2004; Okamura et al., 2008; Radzisheuskaya et al., 2013). We reasoned that identifying and studying proteins that interact with these core pluripotency factors during PGC cell fate determination should reveal new regulators of germ line development and help improve *in vitro* PGC derivation.

Through a genome-wide protein-protein interaction screen using human POU class 5 homeobox 1 (POU5F1 or OCT4), NANOG, SRY-box transcription factor 2 (SOX2), and Kruppel like factor 4 (KLF4) as baits and the arrayed bi-molecular fluorescence complementation (BiFC) platform (Lee et al., 2011), we singled out candidate proteins that could interact with multiple pluripotency factors and examined them in a secondary EpiLC induction screen in reporter mESCs, which uncovered BEN-domain (BEND/Bend) family proteins as novel pluripotency factor-binding partners that could also regulate germ cell differentiation. We showed that Bend5 worked together with Bend4 to promote EpiLC induction *in vitro*. In addition, ATAC-seq analysis indicates that Bend4 and Bend5 did not bind open chromatin. Our findings suggest that BEND/Bend proteins represent a new family of transcriptional modulators and chromatin boundary factors that participate in genes expression regulation during early germline development.

## RESULTS

### A coupled genome-wide screening strategy using BiFC and germ cell differentiation assays

**Figure 1:**
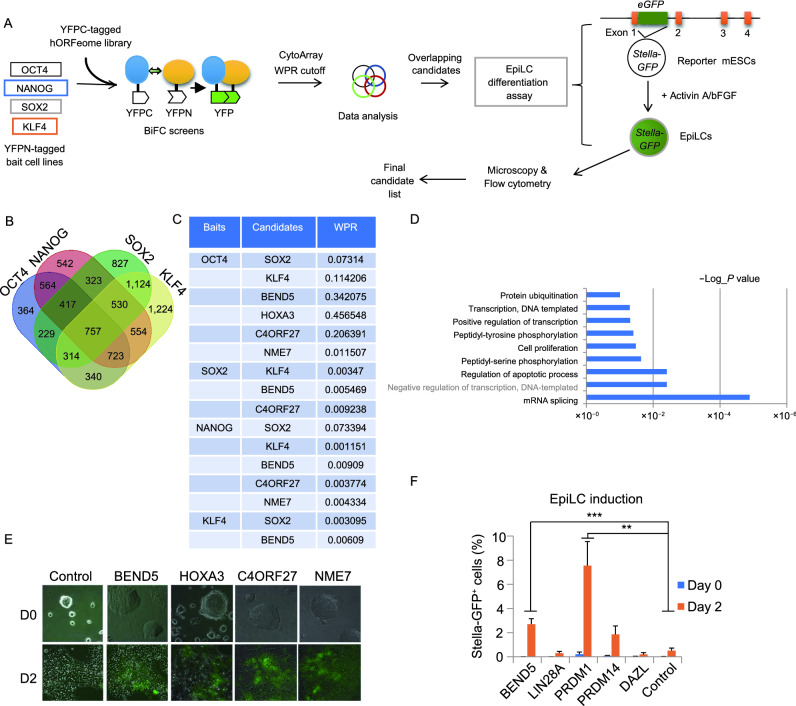
**Coupled BiFC and EpiLC induction screens identify BEND5 as an interacting partner of pluripotency factors and regulator of PGC cell fate**. (A) An arrayed BiFC screen was carried out using HTC75 cells expressing YFPN-tagged baits and YFPC-tagged human ORFeome library. Following flow cytometry analysis and CytoArray data processing, potential interacting proteins of each bait were ranked based on weighted positive ratios (WPR). Overlapping candidates (≥2 baits) were then individually expressed in *Stella-GFP* reporter mESCs for EpiLC induction. Induced cells were examined on day 2 by flow cytometry and microscopy to identify candidates that could upregulate *Stella-GFP* expression. (B) The Venn diagram shows pairwise comparisons of overlapping candidates for each bait in the BiFC screen. (C) A sample list of high-ranking BiFC screen candidates with their WPR values. (D) Gene Ontology (GO) analysis was performed on the biological processes enriched in the 93 overlapping BiFC screen candidates. (E) *Stella-GFP* reporter mESCs ectopically expressing the indicated candidate genes were cultured in EpiLC induction medium and examined by microscopy after two days. Superimposed brightfield and fluorescence images are shown. Empty vector-expressing cells were used as controls. (F) *Stella-GFP* reporter mESCs stably expressing human BEND5 were cultured in EpiLC induction medium and analyzed by flow cytometry after two days. Genes that promote EpiLC and PGCLC nduction (*PRDM1* and *PRDM14*) or known to be PGC markers (*LIN28A* and *DAZL*) were also expressed in the reporter mESCs as controls. Cells expressing vector alone served as negative controls. Error bars represent mean ± SD, *n* = 3 independent experiments. Significance was determined using two-tailed *t* test. ** *P* < 0.01, ***P < 0.001

To better understand the protein interaction networks of core pluripotency factors and identify possible new regulators of PGC cell fate, we developed a coupled screening strategy utilizing the yellow fluorescent protein (YFP)-based BiFC assay (Hu and Kerppola, 2003) and the EpiLC differentiation assay using *Stella-GFP* reporter mESCs (Payer et al., 2006; Hayashi et al., 2011). The BiFC assay enables detection of pairwise protein-protein interactions that bring YFP N terminal (YFPN) and C terminal (YFPC) in two separate proteins to close proximity and allow for their co-folding into a functional fluorescent protein in live cells (Lee et al., 2011). Co-expression of the YFPN and YFPC fragments alone has extremely low YFP background signals (Lee et al., 2011). The YFP-based BiFC assay has been successfully used to map the interactomes governing telomere maintenance (Lee et al., 2011). To carry out the screen, we first generateÔd bait HTC75 cell lines stably expressing YFPN tagged human OCT4, NANOG, SOX2, or KLF4 and then transduced these cells with retroviruses encoding the human ORFeome library fused to YFPC (Fig. 1A). YFP fluorescence complementation was subsequently analyzed by flow cytometry and the CytoArray program to derive candidate gene lists for each bait (Lee et al., 2011). Because the four bait proteins are known to interact with each other, each candidate was further ranked based on its appearance in all four screens to derive a list of overlapping candidates (Fig. 1B). Since many of the human candidate genes share high sequence and functional homology with their mouse counterparts, the top overlapping candidate genes were subsequently stably expressed in *Stella-GFP* reporter mESCs for verification using the EpiLC differentiation assay (Fig. 1A). In these reporter cells, the sequences encoding eGFP were inserted into the first exon of the mouse *Stella*/*Dppa3* locus. Following EpiLC induction, upregulation of Stella expression would allow eGFP to be expressed and detected (Nakaki et al., 2013). Candidate genes that enabled GFP expression during EpiLC induction were thus identified by microscopy and flow cytometry (Table S1).

### Identification of interaction networks of pluripotency factors and novel players in PGC cell fate regulation

Several known interacting pairs were among the higher ranked candidates from our arrayed BiFC screens (e.g., SOX2/KLF4 with OCT4, KLF4 with NANOG/SOX2, and SOX2 with KLF4) (Figs. 1C and S1A), validating our protein-protein interaction screening approach. Of the potential interacting partners, some appeared in all four candidate lists. We selected 93 candidates that ranked high and appeared in at least two BiFC datasets (Table S2). Of this list, OCT4/NANOG have the most overlapping candidates (69/93), followed by NANOG/SOX2 (63/93) and OCT4/SOX2 (57/93), further underscoring the interconnectedness of the pluripotency factor interaction networks. Gene ontology analysis revealed transcription, positive regulation of transcription, transcriptional regulation, and mRNA splicing to be among the top categories (Fig. 1D).

Next, the 93 overlapping human candidate genes were individually expressed in the *Stella-GFP* reporter mESCs and assessed for their activity in EpiLC induction assays (Fig. 1A). By day 2 cells overexpressing several genes (i.e., BEN domain-containing protein 5 (BEND5), Homeobox A3 (HOXA3), Chromosome 4 open reading frame 27 (C4ORF27), and NME/NM23 family member (NME7)) had significantly higher GFP signals compared with controls when analyzed by fluorescence microscopy (Fig. 1E) and FACS (Fig. S1B). In the two-step culturing scheme where EpiLCs were further cultured in PGCLC-inducing medium (Hayashi et al., 2012), ectopic expression of these genes also resulted in significantly higher Stella-GFP signals (Fig. S1C). These experiments not only uncovered previously unknown players in PGC cell fate determination but also imply that their interaction with pluripotency factors may be important to this process.

### Bend5 is a novel interacting protein of pluripotency factors and promotes EpiLC induction from mESCs

**Figure 2:**
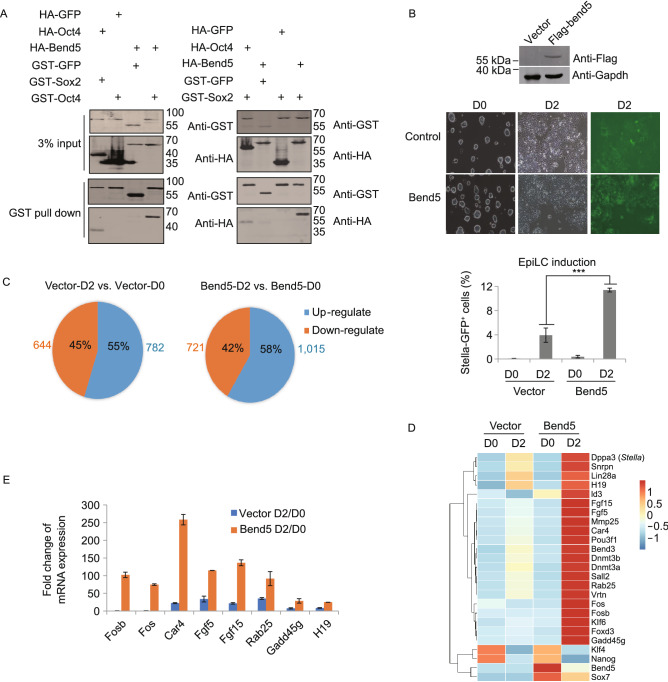
**Mouse Bend5 overexpression promotes EpiLC induction from mESCs and leads to transcriptomic changes**. (A) 293T cells transiently co-expressing HA-tagged mouse Bend5 with GST-tagged mouse Oct4 (left) or mouse Sox2 (right) were analyzed in GST-pulldown assays. The precipitates were resolved by SDS-PAGE and western blotted with the indicated antibodies. Cells co-expressing HA-Oct4 and GST-Sox2 served as positive controls. GST or HA-tagged GFP were used for negative controls. (B) *Stella-GFP* reporter mESCs stably expressing Flag-tagged Bend5 were either western blotted to confirm Bend5 expression (top) or differentiated into EpiLCs (middle) where bright-field and fluorescence microscopy was performed at the indicated time points. Cells expressing vector alone were used as controls. The same cells were also analyzed by flow cytometry (bottom), where the percentage of GFP^+^ cells before and after induction was calculated. Error bars represent mean ± SD, *n* = 3 independent experiments. Significance was determined using two-tailed *t* test. ***P < 0.001. (C) Based on RNA-seq data using control and Bend5-expressing mESCs collected from EpiLC induction assays, the number of genes with >2-fold change following induction was plotted as shown. (D) Based on RNA-seq data, a select list of genes differentially up- or down-regulated following EpiLC induction in control vs. Bend5-expressing cells were plotted in the heatmap. (E) Stella-GFP reporter mESCs stably expressing vector alone or Bend5 were induced to differentiate into EpiLCs. GFP positive cells were FACS sorted at day 0 (D0) or 2 (D2) for RT-PCR analysis. Fold change (D2/D0) was calculated and plotted as shown. *Fosb* and *Fos* function in the TGFβ signaling pathway. *Car4* encodes a metabolic enzyme. *Fgf5* and *Fgf15* are epiblast-specific genes. *Rab25*, *Gadd45g*, and *H19* are developmentally regulated genes. Error bars represent mean ± SD, *n* = 3 independent experiment

Named for its presence in BANP/E5R/NAC1, the BEN domain is a highly conserved bioinformatically defined motif (Abhiman et al., 2008). BEN domain-containing proteins can be found from *Drosophila* to mammals and have been implicated in chromatin regulation during early development (Dai et al., 2013). In our BiFC screens, BEND5 was not only identified from all four baits but also one of the highest ranked interaction candidates for OCT4, NANOG, and SOX2 (Figs. 1C and S1A). It was very potent in EpiLC-induction assays, at levels comparable to known PGC-promoting factors such as PRDM1/BLIMP1 and PRDM14 (Fig. 1F). In mouse, *Bend5* appeared to be highly expressed in PGCs as well as in tissues such as testis (Fig. S2A). When we examined mouse Bend5 in GST pulldown and pairwise BiFC assays, we confirmed that it indeed could interact with mouse Oct4 and Sox2 (Figs. 2A and S2B). When *Stella-GFP* reporter mESCs stably expressing mouse *Bend5* were cultured in EpiLC-inducing medium, increased GFP expression was observed as well (Fig. 2B). And RT-qPCR analysis of the GFP-positive cells confirmed that increased *Bend5* expression was accompanied by increased *Stella* messages and decreased *Oct4* expression as the cells differentiated (Fig. S2C and S2D). In embryoid body (EB) formation assays, mESCs can aggregate and spontaneously differentiate into cells of different germ layers, including PGC-like cells at relatively low frequency (Wei et al., 2008). When the Bend5-expressing reporter mESCs were examined in EB formation assays, increased Stella-GFP positive (SG^+^) cells were detected compared to controls (Fig. S2E), indicating that overexpressing Bend5 could indeed upregulate *Stella* expression during differentiation.

During early germ cell development, Prdm1/Blimp1 was activated first in a few lineage-restricted PGCs, which then became double positive for both Blimp1 and Stella (Ohinata et al., 2005). To better probe the activity of Bend5, we took advantage of the dual-reporter mESC line expressing *Blimp1-mVenus* (BV) and *Stella-ECFP* (SC), where a small number of double-positive (BVSC^+^) cells could be detected following EpiLC induction (Hayashi et al., 2011, 2012; Nakaki et al., 2013). When Bend5 was stably overexpressed in the dual-reporter cells, increased BVSC^+^ cells were seen in both EpiLC induction and EB formation assays (Fig. S2F). More importantly, RNAi-mediated knockdown of *Bend5* resulted in a decrease in BVSC^+^ cells (Fig. S2G). These data together support the notion that Bend5 may facilitate *in vitro* early germ cell differentiation of mESCs.

### Bend5 overexpression upregulates gene expression in mESCs

Given the apparent role of Bend5 in promoting EpiLC differentiation and gene transcription, we decided to investigate by RNA-seq possible transcriptomic changes in Bend5-overexpressing *Stella-GFP* mESCs that were induced to differentiate. Compared with control cells, ectopic expression of Bend5 led to an increase in the number of genes up-regulated upon differentiation induction (fold change > 2) (Fig. 2C), including canonical primed epiblast cell markers such as *Fgf5* and *Fgf15* (Fig. 2D), as confirmed by RT-qPCR using FACS-sorted GFP^+^ cells (Fig. 2E). Bend5-expressing cells also showed a corresponding drop in the number of genes down-regulated during differentiation (Fig. 2C), such as pluripotency genes *Klf4* and *Nanog* (Fig. 2D and 2E). TGFβ/Activin signal pathways are critical to PGC development *in vitro* and act primarily through SMADs (Duggal et al., 2013, 2015; Yakhkeshi et al., 2018). Smad3/Smad4 can cooperate with AP-1 (c-Jun/c-Fos) to mediate TGFβ-induced transcription (Zhang et al., 1998). Consistent with these findings, *Fos* appeared highly expressed in PGCs compared to MEFs and mESCs (Fig. S2H). Upon EpiLC induction, cells overexpressing Bend5 exhibited increased expression of *Fosb* and *Fos* as well as metabolic enzyme (e.g., *Car4*) and developmental genes (e.g., *Rab25*, *Gadd45γ*, and *H19*) (Fig. 2D and 2E) (Table S3). These results combined implicate Bend5 in promoting EpiLC induction through upregulating genes associated with primed epiblast cells and the TGFβ/Activin signaling pathway. Interestingly, we found a collection of genes that specifically expressed in spermatogonial stem cells (SSCs) were upregulated in Bend5 overexpressed cells compared with control cells, including *Usp26* (*Ubiquitin specific peptidase 26*), *Thy1* (*Thy-1 cell surface antigen*), Sox3 (*SRY-box transcription factor 3*) (Table S4). These results suggested that Bend5 overexpression might promote germ cell differentiation *in vitro*.

### Bend5 regulates DNA demethylation by recruiting Tet1 during EpiLC induction

**Figure 3:**
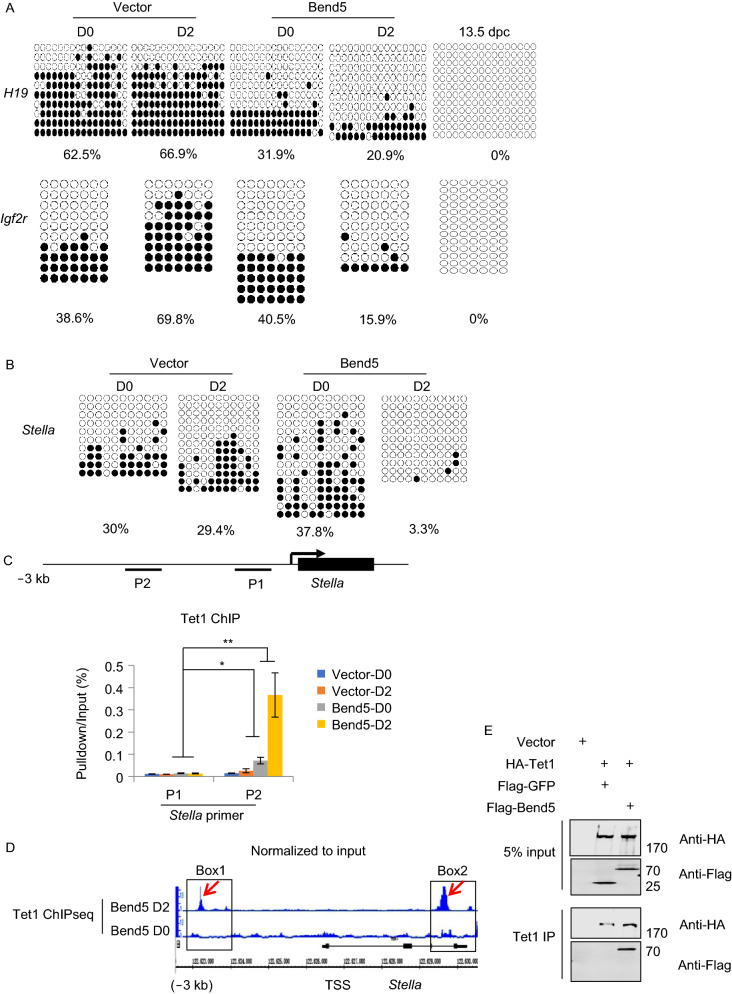
**Bend5 overexpression promotes demethylation at the Stella locus during EpiLC induction**. (A and B) Flag-Bend5-expressing *Stella-GFP* mESCs were induced to differentiate to EpiLCs for two days. At day 0 and 2, GFP positive cells were FACS sorted for genomic DNA extraction and bisulfite sequencing to assess the CpG methylation status of the *H19* and *Igf2r* loci (A) or the *Stela* DMR region (B). Vector alone mESCs and/or 13.5 dpc PGCs served as controls. White and black circles represent unmethylated and methylated CpG sequences respectively. The percentage of methylated CpG sites in the assessed region is listed under each panel. (C) Control and Flag-Bend5-expressing *Stella*-GFP mESCs were induced to differentiate to EpiLCs and harvested (at day 0 and 2) for ChIP analysis using an anti-Tet1 antibody. Two primer sets against regions upstream of *Stella* TSS were designed for real-time PCR. Results were normalized to input. Error bars represent mean ± SD; *n* = 3 independent experiments. Significance was determined using two-tailed *t* test. *P < 0.05, ** *P* < 0.01. (D) mESCs stably expressing Flag-tagged Bend5 were cultured in EpiLC induction media and harvested at day 0 and 2 for ChIP-seq analyses using antibodies against Tet1. Boxed areas indicate regions upstream (Box1) or along the gene body (Box2) of *Stella*. Red arrows indicate Tet1 binding sites. (E) 293T cells co-expressing HA-Tet1 with Flag-Bend5 were harvested for co-immunoprecipitations (co-IP) using an anti-Tet1 antibody. The precipitates were resolved by SDS-PAGE and western blotted with the indicated antibodies. Flag-GFP served as a negative control

It has been well established that PGC development is often accompanied by demethylation of differentially methylated regions (DMRs). For instance, the methylation of imprint genes appears to be erased in 13.5 dpc PGCs (Hajkova et al., 2002; Lee et al., 2002). Activin A treatment can upregulate *Stella in vitro* and inhibiting DNA demethylases Tet1 or Tet2 can downregulate *Stella* expression in mESCs, suggesting that DNA methylation is key to *Stella* expression (Duggal et al., 2013, 2015; Mulholland et al., 2020). The findings of upregulated gene expression in Bend5-overexpressing cells led us to further examine the effects of Bend5 overexpression on DNA methylation in mESCs. We first carried out bisulfite sequencing to analyze the DNA methylation status of imprint genes during EpiLC induction in *Stella-GFP* reporter mESCs expressing Flag-Bend5. As reported before, DNA methylation on *H19* and *Igf2r* were absent in control 13.5 dpc PGCs (Fig. 3A) (Lucifero et al., 2002; Kagiwada et al., 2013). By bisulfite sequencing, we observed that the methylation level in *H19* and *Igf2r* decreased as mESCs passaged in Lif and 2i on feeder-free conditions (called 2i/L mESCs), but not in serum and Lif on feeder cells (called S/L mESCs) conditions (Fig. S3A). In particular, the methylation levels of *H19* and *Igf2r* in 2i/L mESCs at passage P1 and P3 were unchanged, while P11 or P16 showed decreased methylation (Fig. S3B). Furthermore, we observed that Bend5 overexpression did not affect imprint methylation (*H19* and *Igf2r*) at P1 compared with control cells (Fig. S3C), but decreased imprint methylation (*H19* and *Igf2r*) at P3 during EpiLC induction (Fig. 3A). During EpiLC induction, Bend5 overexpression decreased *H19* methylation at D0 by nearly 2 fold (62.5% to 31.9%) but not *Igf2r* (38.6% to 40.5%) compared with control (Fig. 3A). The result indicated that the demethylation of *H19* was partially dependent on the overexpression of Bend5. Upon EpiLC induction, vector alone cells showed minimal change in DNA methylation at the *H19* locus (62.5% to 66.9%). In contrast, *H19* methylation decreased by nearly 3 fold (66.9% to 20.9%) in Bend5-expressing cells when analyzed two days after induction. Similarly drastic reduction of DNA methylation also occurred at the *Igf2r* locus (69.8% to 15.9%) in Bend5-expressing cells after induction (Fig. 3A). These data indicate that Bend5 likely promoted DNA demethylation of imprinted genes during EpiLC differentiation. Next, we examined the methylation status of CpG islands upstream of *Stella*. Again, the level of CpG island methylation in control cells remained essentially unchanged after differentiation, but decreased significantly in Bend5-expressing cells (Fig. 3B).

The dramatic decrease in DNA methylation with Bend5 overexpression suggests involvement of DNA demethylases. Given that Tet1/Tet2 can be targeted to within −2~3 kb upstream of *Stella* (Mulholland et al., 2020), we investigated Tet1/Tet2 occupancy in this region of the *Stella* locus by ChIP-qPCR (chromatin immunoprecipitation and real-time polymerase chain reaction) using mESCs stably expressing Flag-Bend5. Before induction (D0), these cells already exhibited a slight enrichment of Tet1 over control cells (Fig. 3C). After two days of EpiLC induction, Tet1 occupancy at the P2 position increased dramatically (Fig. 3C). In comparison, we found no specific enrichment of Tet2 at position P2 before or after EpiLC induction in Bend5-expressing cells (Fig. S3D). To better explore the enrichment of Tet1 at the *Stella* locus during EpiLC induction, we performed ChIP-seq (chromatin immunoprecipitation followed by sequencing) analyses using *Stella-GFP* mESCs expressing Flag-tagged Bend5. Consistent with the ChIP-qPCR results, in uninduced cells, a slight enrichment of Tet1 could be seen in regions upstream of TSS (transcription start site) (Box1) as well as the gene body (Box2) (Fig. 3D). Following induction, the amplitude of some of these Tet1-binding peaks increased dramatically (red arrows in Box 1 and 2). Notably, Tet1 expression in induced EpiLCs appeared to have decreased relative to D0 mESCs (Fig. S3E), suggesting that the enrichment was likely not a result of increased Tet1 levels. Tet1 was highly expressed in PGCs and responsible for active DNA demethylation of imprints and germline-specific genes (Vincent et al., 2005; Williams et al., 2011; Pastor et al., 2013; Zhang et al., 2016). Notably, dysfunction of Tet1 protein caused hypermethylation at the imprints (*H19*, *Igf2r*, *Peg1*, *Mest* etc.) and Tet1 knockout also led to hypermethylation at the imprints (*H19* and *Igf2r*) (Liu et al., 2015; Zhang et al., 2016). These studies suggested that Tet1 indeed negatively regulated DNA methylation level of imprint genes. We speculated that Bend5 might promote DNA demethylation by actively recruiting Tet1 to the *Stella* promoter during EpiLC induction.

To test this idea, we co-expressed Flag-Bend5 and HA-tagged Tet1 in 293T cells and examined their interaction. As shown in Fig. 3E, Bend5 could indeed co-immunoprecipitate (co-IP) with Tet1. Our data thus far support the hypothesis that upon EpiLC differentiation, Tet1 may target to the *Stella* locus (and possibly other DMRs) through interaction with Bend5, thereby promoting DNA demethylation and activating gene transcription.

### Bend5 binds to the promoter region of *Stella* through its BEN domain

**Figure 4:**
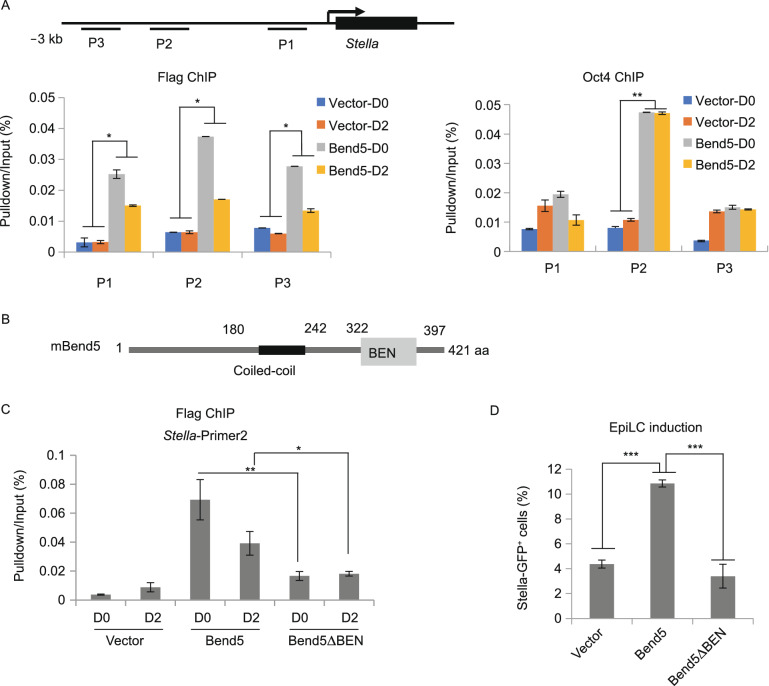
**Bend5 binds to the promoter region of the Stella gene through its BEN domain**. (A) Control and Flag-Bend5-expressing mESCs were induced to differentiate into EpiLCs. Cells were harvested at day 0 and 2 for ChIP assays using anti-Flag (left) and anti-Oct4 (right) antibodies. Three primer sets against regions upstream of *Stella* TSS were designed for real-time PCR. Results were normalized to input and graphed as shown. Error bars represent mean ± SD, *n* = 3 independent experiments. Significance was determined using two-tailed *t* test. *P < 0.05, **P < 0.01. (B) Mouse Bend5 contains a coiled-coil domain and a BEN domain. (C and D) *Stella-GFP* mESCs stably expressing Flag-tagged full-length Bend5 or the BEN domain deletion mutant Bend5ΔBEN were induced to differentiate into EpiLCs. Cells expressing vector alone served as controls. Cells were harvested at day 0 and 2 for ChIP-qPCR analysis using an anti-Flag antibody and primer set 2 from above (C) or flow cytometry to obtain the percentage of *Stella-GFP* positive cells (D). ChIP-qPCR results were normalized to input. Error bars represent mean ± SD, *n* = 3 independent experiments. Significance was determined using two-tailed *t* test. *P < 0.05, ** P < 0.01, *** P < 0.001

Oct4 has been reported to bind the ATTTGCAT sequence in the *Stella* promoter region (−1.74 kb) for stem cell pluripotency maintenance (van den Berg et al., 2008). Given the interaction between Bend5 and Oct4 and the ability of BEN domains to bind DNA in a sequence-specific manner (Dai et al., 2015, 2013), we hypothesized that in addition to recruiting DNA demethylases, Bend5 might also directly target to the *Stella* locus to upregulate *Stella* expression. To test this idea, we first examined Bend5 occupancy in the promoter region of *Stella* by ChIP using Flag-Bend5 expressing mESCs. Before EpiLC induction, significant enrichment of Bend5 could be detected in Bend5-expressing cells at several positions upstream (within ~3 kb) of *Stella* TSS compared with control cells (Fig. 4A). Once cells were cultured in EpiLC induction medium, Bend5 enrichment at the *Stella* locus became less pronounced but remained significantly higher than in control cells two days after induction (Fig. 4A), even though expression of Bend5 itself had been drastically downregulated by this time (Fig. S2C), supporting the notion that Bend5 could target directly to the *Stella* promoter. Moreover, these Bend5-binding sites appeared to overlap with those of Oct4 (Fig. 4A) (van den Berg et al., 2008). In fact, Bend5 overexpression also increased Oct4 occupancy on *Stella*, suggesting that co-occupancy of Oct4 and Bend5 may be important for *Stella* upregulation.

When we compared *Stella-GFP* reporter mESCs expressing full-length Bend5 vs. the BEN domain deletion mutant (Bend5∆BEN), we found much lower Bend5 enrichment at the *Stella* locus in Bend5∆BEN-expressing cells (Fig. 4B and 4C), even though Bend5∆BEN showed similar nuclear localization as full-length Bend5 (Fig. S3F). Importantly, BEN domain deletion led to reduced GFP^+^ cells following EpiLC induction (Figs. 4D and S3G). To further probe the DNA-binding activity of Bend5, we performed electrophoretic mobility shift assays (EMSA) using recombinant full-length and BEN domain deletion Bend5 proteins (Figs. 4E and S3H). Multiple DNA oligos corresponding to sequences in the promoter region of *Stella* could be shifted by full-length Bend5 but not Bend5∆BEN, indicating the importance of the BEN domain in mediating Bend5 binding to DNA (Fig. S3I). These data together strongly support a role of Bend5 in regulating *Stella* expression during EpiLC differentiation and underline the importance of the BEN domain to Bend5 function.

### The BEN domain-containing protein Bend4 can also interact with Oct4 and regulate *Stella* activation

**Figure 5:**
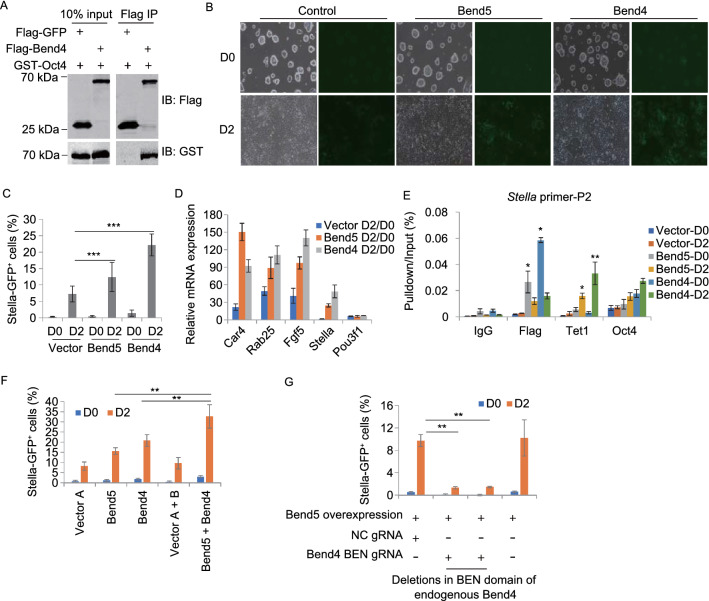
**Bend5 synergizes with Bend4 in induction of Stella expression**. (A) 293T cells co-expressing different combinations of epitope-tagged GFP, Bend4, and Oct4 proteins were harvested for co-IP using an anti-Flag antibody. The precipitates were resolved by SDS-PAGE and Western blotted with the indicated antibodies. (B) *Stella-GFP* mESCs stably expressing Flag-tagged Bend4 or Bend5 were cultured in EpiLC-inducing media. Bright-field and fluorescence imaging was done at day 0 and 2. Vector alone cells served as controls. (C) Cells from (B) were analyzed by flow cytometry to calculate the percentage of GFP^+^ cells at day 0 and 2. Error bars represent mean ± SD; *n* = 3 independent experiments. Significance was determined using two-tailed *t* test. ***P < 0.001. (D) Relative change in mRNA levels of the indicated genes in cells from (B) was determined by RT-qPCR. Error bars represent mean ± SD; *n* = 3 independent experiments. (E) *Stella-GFP* mESCs stably expressing Flag-tagged Bend5 and Bend4 individually were cultured in EpiLC-inducing media and analyzed by ChIP-qPCR using antibodies against the indicated genes at day 0 and 2. Vector alone cells served as controls. IgG served as ChIP analysis controls. Primer set 2 of the *Stella* locus was used for real-time PCR. ChIP-qPCR results were normalized to input. Error bars represent mean ± SD; *n* = 3 independent experiments. Significance was determined using two-tailed *t* test. *P < 0.05, ** *P* < 0.01. (F) *Stella-GFP* mESCs stably expressing Flag-tagged Bend5 and Bend4 together were cultured in EpiLC-inducing media and analyzed by flow cytometry to calculate the percentage of GFP^+^ cells at day 0 and 2. Cells expressing vector alone or Bend5 and Bend4 alone served as controls. For cells co-expressing Bend4 and Bend5, two different vector backbones were used to express the genes and together served as controls. Error bars represent mean ± SD; *n* = 3 independent experiments. Significance was determined using two-tailed *t* test. ** *P* < 0.01. (G) The cell clones carried deletions in BEN domain of endogenous Bend4 from *Stella-GFP* reporter mESCs that also stably expressed Flag-Bend5 were cultured in EpiLC-inducting media and analyzed by flow cytometry to calculate the percentage of GFP^+^ cells at day 0 and 2. Cells generated using non-targeting gRNAs (gRNA-NC) and parental Flag-Bend5-expressing *Stella-GFP* reporter mESCs served as controls. Error bars represent mean ± SD; *n* = 4 independent experiments. Significance was determined using two-tailed *t* test. **P < 0.01

Of the four BEN-domain family members present in the ORFeome library (BEND3, BEND5, BEND6, and BEND7), only BEND5 met all the criteria we set for the coupled screen. When we examined the relative mRNA levels of several BEN-domain family members, we found high expression of *Bend4* in not only PGCs and mESCs but also testis (Fig. S4A and S4B). Of the two Bend4 isoforms, only isoform 1 contains the BEN domain and appeared to be the predominant isoform in mESCs (Fig. S4C). We therefore focused on this longer isoform for this study. Similar to Bend5, Flag-tagged mouse Bend4 could also co-IP with GST-tagged Oct4 (Fig. 5A). When *Stella-GFP* reporter mESCs overexpressing Bend4 were induced to differentiate into EpiLCs, there were more GFP^+^ cells as well (Figs. 5B, 5C and S4D), with upregulated expression of epiblast cell-related genes such as *Fgf5*, *Car4*, and *Rab25* and increased Oct4 and Tet1 binding to the *Stella* locus (Fig. 5D and 5E). These results combined support the notion that Bend4 is another BEN-domain family member that can interact with pluripotency factors and participate in regulating gene expression during PGC development.

### Bend5 synergizes with Bend4 in induction of *Stella* expression

To further probe the relationship between Bend5 and Bend4, we next carried out EpiLC induction assays using *Stella-GFP* reporter mESCs co-expressing Bend4 and Bend5 (Fig. S4D and S4E). While cells ectopically expressing either Bend5 or Bend4 showed comparable increase in GFP^+^ cells following induction, Bend4/Bend5 co-expression resulted in more GFP^+^ cells than expression of either gene alone (Fig. 5F), indicating possible synergy and non-redundant functions of the two genes. Compared to Bend5, Bend4 appeared to be expressed at much higher levels in mESCs than in MEFs (Figs. S2A and S4A). We therefore decided to target *Bend4* in Bend5-expressing *Stella-GFP* reporter mESCs using the CRISPR/Cas9 technology with a sgRNA that targets the Bend4 BEN domain (Fig. S4F). Two clones with deletions in the BEN domain were isolated. As shown in Fig. 5G, disrupting the Bend4 BEN domain led to markedly reduced numbers of GFP^+^ cells, suggesting that Bend5 regulation of *Stella* expression was dependent on the BEN domain of Bend4. The above findings support the model in which Bend5 and Bend4 facilitate transcriptional activation of the *Stella* gene by cooperating with Oct4 and Tet1 in response to EpiLC induction signals.

### Bend5 co-occupies PGC-related genes with Bend4

**Figure 6:**
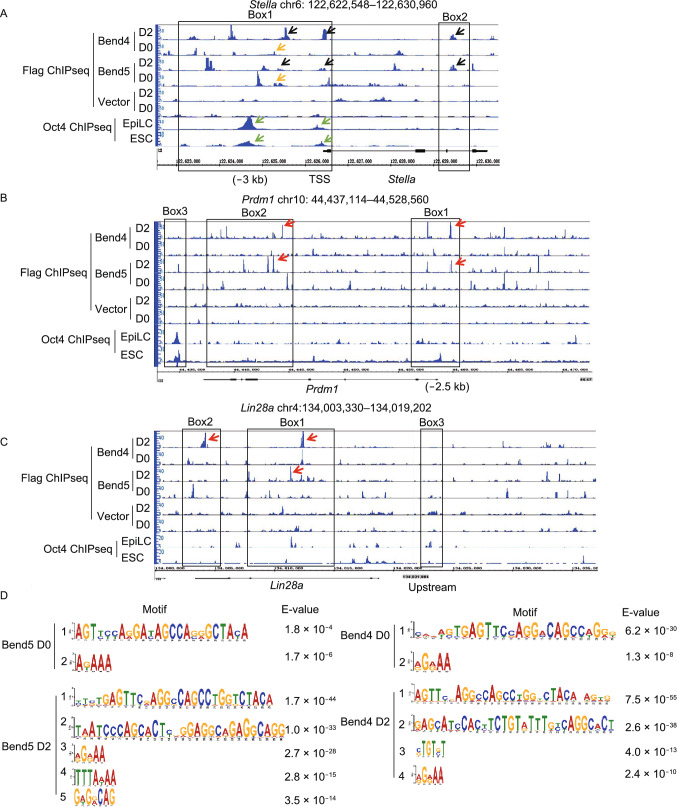
**Bend4 and Bend5 can target to PGC-related genes**. (A) mESCs stably expressing Flag-tagged Bend4 or Bend5 were cultured in EpiLC induction media and harvested at day 0 and 2 for ChIP-seq analyses using antibodies against Flag. Oct4 peaks were based on previously published ChIP-seq data (Buecker et al., 2014). Different arrows indicate binding sites of various proteins. Boxed areas indicate regions upstream (Box1) or along the gene body (Box2) of *Stella*. (B and C) Bend4 and Bend5 binding peaks based on anti-Flag ChIP-seq data from (A) were compared to Oct4 ChIP-seq data (Buecker et al., 2014) for the *Prdm1* (B) and *Lin28a* (C) gene loci. Different arrows indicate Bend4 and Bend5 peaks at day 2. (D) Consensus binding motifs of Bend4 and Bend5 at day 0 and 2 upon EpiLC induction were derived using MEME (for long motif, ~30 bp) or DREME (for short motif, ≤10 bp)

To better define the activities of Bend5 and Bend4 and their target genes during EpiLC induction, we performed ChIP-seq analyses using *Stella-GFP* mESCs individually expressing Flag-tagged Bend5 or Bend4. At the *Stella* locus, the overall binding patterns of Bend5 and Bend4 showed some overlapping peaks but were not identical (Fig. 6A, D0 orange arrows and D2 black arrows). A noticeable increase in both the number and amplitude of Bend5/Bend4 binding peaks could be detected by day 2 of EpiLC induction (Fig. 6A, Box 1), which is consistent with our findings in the region upstream of *Stella* TSS (−3 kb). Within the gene body of *Stella*, there were obvious changes as well in the location and amplitude of Bend5/Bend4 binding peaks following differentiation induction (Fig. 6A, Box 2). Based on published Oct4 ChIP-seq data using mESCs induced to differentiate into EpiLCs by Activin A (Buecker et al., 2014), increased Oct4 binding to the −1.74 kb and TSS regions of the *Stella* gene could be observed by day 2. These peaks partially overlap with those of Bend5 and Bend4 (Fig. 6A, green arrows), which is consistent with our ChIP-qPCR results (Fig. 4A) and further supports the idea that Bend5/Bend4 can co-occupy the *Stella* locus with Oct4.

In addition to *Stella*, Bend5/Bend4 binding could also be detected at the loci of transcription factors *Prdm1*, *Prdm14*, *Tfap2c*, and *Lin28a* (Figs. 6B, 6C, S5A and S5B), which have been shown to promote PGC development (Kurimoto et al., 2008a, 2008b; Yamaji et al., 2008). The binding peaks were distributed in the gene body as well as regions upstream of TSS. Some of these peaks (red arrows) also increased in amplitude following EpiLC induction (Figs. 6B, 6C, Box 1 and 2; S5A and S5B). Again, we found overlap in binding peaks with Oct4 on *Prdm1* and *Lin28a* (Fig. 6B and 6C, Box 3), adding additional support to the notion that Bend5/Bend4 can cooperate with Oct4 on genes important for PGC development.

### Bend5 and Bend4 dynamically associate with DNA during EpiLC induction

Individual BEN domains can vary greatly in actual sequences and consensus binding motifs (Dai et al., 2015). For instance, motif analysis by CentriMo predicts quite distinct consensus motifs for Bend5 and Bend4 (either before or after induction) compared to the *Drosophila* Bend protein Insv, which functions as a transcriptional repressor in neural development (Fig. S5C) (Bailey and Machanick, 2012; Dai et al., 2013). Our ChIP-seq data indicate partial overlap (<20%) of Bend5/Bend4-binding sites throughout the genome (Fig. S5D). The BEN domains of Bend5 and Bend4 share 26% sequence identity and 44% similarity (Fig. S5E), which may help explain their overlapping but different DNA-binding patterns in cells and support the idea that these two genes can function both collaboratively and independently during germ cell development. Results from the EM-based MEME (long motifs of ≤30 bp in length) and novel word-based DREME (shorter motifs of ≤8 bp) algorithms for *de novo* motifs suggest somewhat similar binding motifs for Bend5/Bend4 before differentiation (Fig. 6D) (Bailey, 2011). Differentiation induction not only increased the number of predicted motifs but also altered the specific motif sequences, although motif #1 remained largely unchanged and all of the predicted motifs can be found at the *Stella* locus (Fig. S5F). These alterations signal the dynamic nature of Bend5/Bend4 binding to their target sites during EpiLC differentiation. Coupled with their apparent abilities to elevate enrichment of Tet1 and Oct4 at the *Stella* locus, these results also hint at chromatin re-organization that resulted from Bend5/Bend4 overexpression.

**Figure 7:**
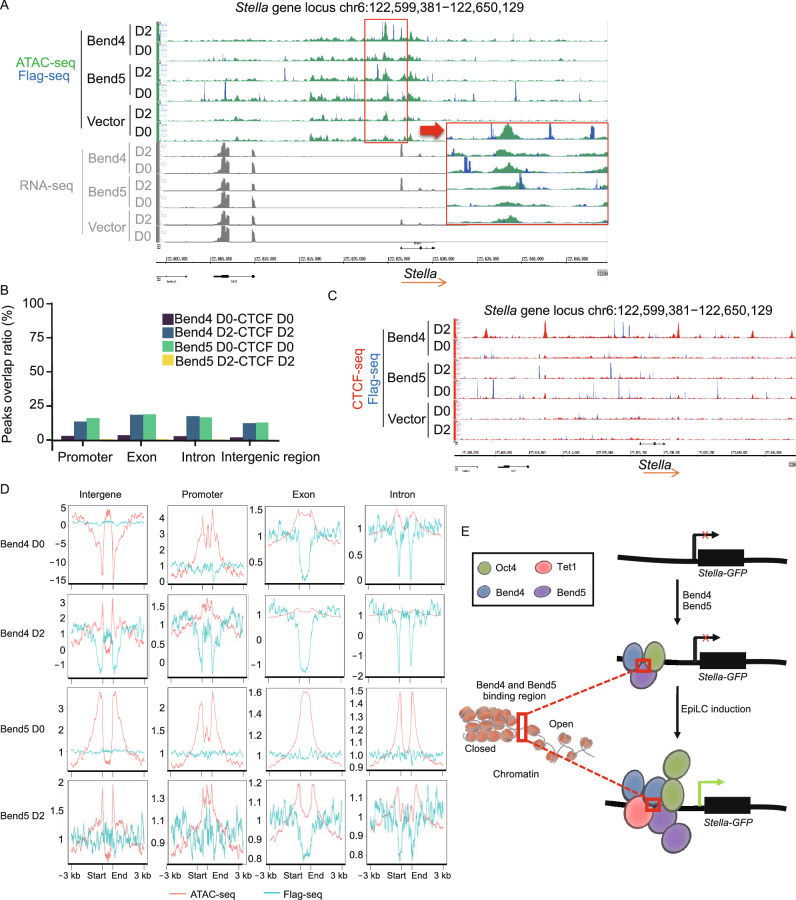
**Bend4 and Bend5 are new chromatin boundary factors**. (A) mESCs stably expressing Flag-tagged Bend5 or Bend4 were cultured in EpiLC induction media and harvested at day 0 and 2 for ATAC-seq. Binding peaks from ATAC-seq were superimposed with binding peals from Flag ChIP-seq data for regions neighboring the *Stella* gene. As comparison, *Stella* mRNA levels from RNA-seq data were plotted below. Blue, Flag ChIP-seq peaks. Green, ATAC-seq peaks. Areas inside red box in the middle were enlarged and shown on the right. (B) mESCs stably expressing Flag-tagged Bend4 or Bend5 were cultured in EpiLC induction media and harvested at day 0 and 2 for ChIP-seq analyses using anti-CTCF antibody. The percentage of overlapping peaks in various genomic regions between Flag and CTCF ChIP-seq before and after induction was calculated and graphed as shown. (C) CTCF binding peaks were superimposed with those of Flag-Bend5/Bend4 for regions adjacent to the Stella gene. Red, CTCF ChIP-seq peaks. Blue, Flag ChIP-seq peaks. (D) Unique Bend4 and Bend5 binding peaks were derived by comparison with control cells and then superimposed with ATAC-seq peaks for the indicated genomic regions. Red, ATAC-seq profiles. Blue, Bend4 or Bend5 profiles. (E) Our data support a model in which Bend4, Bend5, and Oct4 can bind to regions upstream of the *Stella* gene in Bend5/Bend4 co-expressing mESCs. EpiLC induction signals lead to increased enrichment of Bend4, Bend5, and Oct4, as well as elevated Tet1 occupancy for DNA demethylation and *Stella* gene expression activation. At the same time, Bend4 and Bend5 do not binding to open chromatin and mark the boundary of open and closed chromatin on the *Stella* locus, ultimately activating *Stella* gene expression

To assess globally how chromatin accessibility might change in mESCs stably expressing Bend5/Bend4, we decided to employ ATAC-seq (assay transposase-accessible chromatin with high-throughput sequencing) (Buenrostro et al., 2013) and performed peak overlapping analyses using data from ATAC-seq and ChIP-seq for Bend5/Bend4-binding sites. We found very little overlap (<5%) between Bend5/Bend4-binding sites and ATAC-seq peaks throughout the genome with or without EpiLC induction (Fig. S6A), suggesting a lack of Bend5/Bend4 binding to accessible chromatin. When we superimposed ATAC-seq and Flag ChIP-seq peaks at the *Stella* locus, we found Bend5/Bend4-binding peaks to concentrate near the boundaries of open and closed chromatin (Fig. 7A, red box). Similar patterns were also apparent on other PGC-related genes (e.g., *Prdm14*, *Lin28a*, and *Tfap2c*) (Fig. S6B). When the data were cross-referenced with our RNA-seq results for the *Stella* locus, it was clear that Bend5 and Bend4 significantly promoted the expression of *Stella* exon 1 and exon 2 compared to controls, with concentrated ATAC-seq and Flag ChIP-seq peaks in this region. These observations indicate that Bend5 and Bend4 might act as boundary factors and collectively contribute to *Stella* expression upon EpiLC induction.

### Bend5 and Bend4 are a new family of chromatin boundary factors

The organization of genomes is critical to genome replication and gene expression. Eukaryotic chromosomes have been found to fold into domains where intra- and inter-domain interactions can occur through contact regions known as topologically associating domains, which are evolutionarily conserved features and formed with the help of special elements called chromatin boundaries or insulators that are capable of pairing with each other (Beagan and Phillips-Cremins, 2020; Dixon et al., 2016, 2012; Pope et al., 2014). Boundary element pairing may be achieved through the action of sequence-specific DNA-binding proteins such as the CCCTC-binding factor (CTCF) and their co-factors. CTCF is a multi-functional ubiquitously expressed protein with 11 tandem C2H2 zinc fingers (Lobanenkov et al., 1990; Fischer et al., 1993). It functions in transcriptional regulation and genome organization (Gaszner and Felsenfeld, 2006; Wendt et al., 2008; Handoko et al., 2011; Plasschaert et al., 2014). CTCF-null embryos could not implant and died by pre-implantation (Wan et al., 2008; Moore et al., 2012), and conditional CTCF KO mice were infertile with a 90% reduction in sperm count (Hernandez-Hernandez et al., 2016). CTCF can homodimerize and interact with cohesion to mediate long-distance chromatin interaction and plays a critical role in cell type-specific genomic organization and chromatin boundary function (Ji et al., 2016; Beagan et al., 2017; Arzate-Mejia et al., 2018).

To investigate possible interplay between Bend5/Bend4 and CTCF during EpiLC induction, we performed CTCF ChIP-seq analyses using *Stella-GFP* mESCs individually expressing Flag-tagged Bend5 or Bend4. Results from CTCF ChIP-seq were then compared to Flag ChIP-seq data using the same cells (Fig. 7B). Before EpiLC induction, there was minimal overlap (<5%) between CTCF unique peaks and those of either Bend5 or Bend4 throughout the genome (Fig. 7B). The percentage of overlap increased to <20% after EpiLC induction, indicating that the majority of Bend5/Bend4 peaks remained distinct from CTCF peaks. Furthermore, when we overlaid the CTCF peaks with those of Bend5 or Bend4 on the *Stella* locus we found little overlapping as well regardless of EpiLC induction (Fig. 7C). Similar results were obtained when we compared CTCF and Bend5/Bend4 binding on other PGC-related genes (e.g., *Prdm14*, *Lin28a*, and *Tfap2c*) (Fig. S6C).

Whole-genome analysis of CTCF in chromatin boundary regions revealed demarcation of repressive and active chromatin domains (Cuddapah et al., 2009). Given the apparent binding difference between Bend5/Bend4 and CTCF, we analyzed the distribution of Bend5/Bend4-binding sites and ATAC-seq peaks throughout the genome after EpiLC induction. We found reverse correlation between Bend4-binding sites and whole-genome ATAC-seq peaks, and between Bend5-binding sites and exons and introns of ATAC-seq peaks (Fig. 7D), suggesting that Bend4 and Bend5 likely bind to closed chromatin. Our data support the model where Bend5 and Bend4 help mark the boundary of open/closed chromatin after EpiLC induction and activate the transcription of epiblast-related genes during early germ cell differentiation (Fig. 7E). Our study should provide insights into the mechanism of germ cell differentiation and new transcription factor combinations to enhance germ cell generation.

## DISCUSSION

Studying the mechanisms and regulatory factors of germ cell fate specification is necessary to improving the efficiency of *in vitro* germ cell production and developing effective strategies to combat genetic diseases. The understanding of human PGC specification has relied heavily on studies using murine models, such as *in vitro* mouse PGC specification from mESCs (Hayashi et al., 2018). Many transcription factors including several core pluripotency genes are important in both human and mouse PGC development (Kehler et al., 2004; Irie et al., 2015; Fang et al., 2018). For instance, OCT4/Oct4 can target to genes important for PGC development (e.g., *Stella*, *BlIMP1*, *NANOS2*) and is essential for germ cell lineage determination (Kehler et al., 2004; van den Berg et al., 2008; Murakami et al., 2016; Fang et al., 2018). By carrying out genome-wide protein-protein interaction screens of four core pluripotency factors and selecting overlapping candidates for further analysis, we sought to identify genes that promote PGC specification *in vitro*. Although Stella is not essential for PGC development, its upregulation may help maintain DNA hypomethylation in hypomethylated PGCs (Payer et al., 2003; Nakamura et al., 2007; Han et al., 2018, 2019; Li et al., 2018; Mulholland et al., 2020). In this study, we used *Stella* expression as a reporter to assess whether candidates obtained from BiFC screens in human cells could promote EpiLC induction. Several genes proved highly efficient at inducing EpiLC formation when overexpressed in mESCs. In particular, we showed that two BEND/Bend family members—Bend5 and Bend4—are sequence-specific DNA-binding proteins that could synergistically promote EpiLC induction from mESCs. BEND/Bend family proteins are found in a wide array of organisms (Abhiman et al., 2008). Further studies into these new functions of the BEND/Bend family, such as transcriptional control and chromatin boundary demarcation, should provide new insights into the mechanisms of germ cell differentiation and alternative strategies for enhancing human PGC specification and PGC induction *in vitro*.

Germline development undergoes epigenetic reprogramming that includes changes in DNA methylation, histone modification, and chromatin remodeling, which ultimately leads to transcriptional activation/repression (Guibert et al., 2012; Kurimoto et al., 2015; Magaraki et al., 2017; Nakaki et al., 2013). For *in vitro* induction of mouse PGCLCs, EpiLC induction and subsequent generation of *Stella*/*Blimp1* double positive PGCs (Hayashi et al., 2011; Nakaki et al., 2013) are concomitant with DNA demethylation of imprint loci and PGC-related genes (Guibert et al., 2012; Nakaki et al., 2013; Gkountela et al., 2015; Miyoshi et al., 2016; Tang et al., 2016). In previous studies, DNA methylation change on H19 in PGCLCs inducted using Blimp1, Prdm14 and Tfap2c was relatively small compared to EpiLCs (44.7% vs. 37.5%) (Nakaki et al., 2013). Here, we found that overexpression of Bend5 led to dramatic increases in DNA demethylation of both imprint loci (*H19* and *Igf2r*, ~3-fold) and at the *Stella* locus (~10 fold) during EpiLC induction, which were coupled with increased Tet1 recruitment. During somatic cell reprogramming, Tet1 can activate germline regulatory genes (Bartoccetti et al., 2020). In PGCs, Tet1 appears to play a more limited role in DNA demethylation during PGC specification, mainly during late stages of germ cell development (Yamaguchi et al., 2013; Hill et al., 2018). In mESCs, Tet1 may be targeted to the *Stella* locus for active demethylation (Mulholland et al., 2020). In line with this, we observed increased Tet1 recruitment at the *Stella* locus in Bend5 and Bend4-expressing mESCs during EpiLC induction. Collectively, our data support the model that Bend5 and Bend4 along with Tet1 may promote active DNA demethylation during mESC differentiation towards germ cells.

The binding motifs of the BEN domains of Bend5 and Bend4 appear to be different from that of Insv (Dai et al., 2013; Fedotova et al., 2019), which may help explain how they target to different genomic regions and mediate different functions. Bend5 and Bend4 can both target to PGC-related genes, whose binding patterns shift upon EpiLC induction. During PGC specification, expression of germline genes (e.g., *Blimp1* and *T*) appears correlated with low H3K27me3 and high H3K27ac levels, consistent with active/repressed chromatin re-organization (Barski et al., 2007; Kurimoto et al., 2015; Liu et al., 2016; Magaraki et al., 2017). With the global loss of H3K9me2 and DNA demethylation in PGC development, there is a genome-wide change in open/closed chromatin (Kurimoto et al., 2015). We provide evidence here that Bend5 and Bend4 may act as boundary factors of open-closed chromatin and upregulate the expression of genes such as *Stella* after EpiLC induction. Bend5 and Bend4 may thus cooperatively participate in marking dynamic chromatin boundaries during early PGC development. Probing further into the activities and mechanisms of these factors should provide new insights into the interplay between transcriptional control and chromatin boundary in germ cell differentiation.

## MATERIALS AND METHODS

### Vectors, cell lines, and antibodies

cDNAs encoding full-length human BEND5 were cloned into a retrovirus vector (EF1α promoter and Flag-HA c-terminal tag), while mouse Bend5, Bend4, and their truncation mutants were cloned into a lentivirus vector or pDEST27 (Invitrogen) for mammalian expression. cDNAs encoding mouse Oct4 and Sox2 were cloned into a MSCV-based retroviral vector (HA-Flag c-terminal tagging) or pDEST27.

*Stella-GFP* reporter mESCs (gift from Dr. Yuan Wang, East Normal University, China) were maintained on MEF feeder cells in Dulbecco’s modified Eagle’s medium supplemented with 15% fetal calf serum (Hyclone, Logan, UT, US), 0.1 mmol/L NEAA, 1 mmol/L sodium pyruvate, 0.1 mmol/L β-mercaptoethanol, 100 U/mL penicillin, 0.1 mg/mL streptomycin, 2 mmol/L L-glutamine, and LIF (1000 U/mL, Millipore, Billerica, MA). HTC75 and HEK293T cells were cultured in Dulbecco’s modified Eagle’s medium with high glucose supplemented with 15% fetal calf serum (Hyclone, Logan, UT, US).

Antibodies used in this study: anti-Flag (Sigma F7425 for ChIP and ChIPseq; ANTI-FLAG M2 Affinity beads (A2220) for IP, Abmart M20008 (Mouse) and Sigma F7425 (Rabbit) for WB), anti-CTCF (Active Motif, 61311), anti-Oct4 (Santa Cruz, sc5279), anti-Tet1 (Active Motif, 61443), anti-HA (Sigma, H3663), anti-GAPDH (ABclonal, AC027), mouse polyclonal anti-GST (Abmart, M20007), rabbit polyclonal IgG (Millipore, 12-370).

Sequences for Bend5 shRNAs and all primers and oligonucleotide are listed in Table S3.

### Arrayed genome-wide bi-molecular fluorescence complementation (BiFC) screens

The arrayed BiFC screening strategy and data analysis procedures were as previously described (Lee et al., 2011). Briefly, HTC75 cells stably expressing YFPn-tagged (amino acids 1–155 of Venus YFP) bait proteins (OCT4, NANOG, SOX2, or KLF4) were generated and seeded into 96-well plates before being infected with retroviruses encoding YFPc-tagged (amino acids 153–239 of Venus YFP) prey library. Fluorescence complementation was determined by flow cytometry and data were analyzed by CytoArray, which calculates weighted positive ratios (WPR) based on fluorescence signals. Of the potential interacting partners from all four baits (Table S1), 93 candidates were chosen for further analysis (Table S2).

### *In vitro* EpiLC induction and flow cytometry analysis and cell sorting (FACS)

EpiLC induction was performed as previously described (Hayashi et al., 2011). Briefly, mESCs that were maintained on MEF feeder cells in mESC medium were transferred to feeder-free KSR medium (Knock-out Dulbecco’s modified Eagle’s medium with 10% KnockOut™ Serum Replacement (KSR) (ThermoFisher, 10828028, US), 0.1 mmol/L NEAA, 1 mmol/L sodium pyruvate, 0.1 mmol/L β-mercaptoethanol, 100 U/mL penicillin, 0.1 mg/mL streptomycin, 2 mmol/L L-glutamine, LIF and 2i (CHIR99021 3 µmol/L; PD0325901 1 µmol/L)) and cultured for two passages before being induced to EpiLCs with N2B27 medium (KSR (1%), Activin A (20 ng/mL), bFGF (12 ng/mL), and 2i) for two days with medium change daily. EpiLCs were collected and filtered on Day 2 for flow cytometry analysis. FACS sorting was performed with a FACSAria or FACSAriaIII (BD) cell sorter and results were analyzed with FACSDiva (BD) or Flowjo (Tree Star) software.

### Primordial germ cells like cells (PGCLCs) Induction and flow cytometry analysis

PGCLCs induction was performed as previously described (Hayashi et al., 2011). EpiLCs (~10^3^) were seeded in U-bottom 96-well plates with low cell binding surface (NUNC), and cultured in serum-free medium (GMEM [Invitrogen] with 15% KSR, 0.1 mmol/L NEAA, 1 mmol/L sodium pyruvate, 0.1 mmol/L β-mercaptoethanol, 2 mmol/L L-glutamine, LIF (1000 U/mL; Invitrogen), BMP4 (500 ng/mL; R&D Systems), BMP8b (500 ng/mL; R&D Systems), SCF (100 ng/mL; R&D Systems), and EGF (50 ng/mL; R&D Systems) for six days with medium change every day. PGCLCs was collected and filtered, *Stella-GFP* positive or *Blimp1-mVenus* (BV) and *Stella-ECFP* (SC) positive cells were detected with the FITC and AmCyan Horizon V500 channel, respectively. The results were analyzed with FACSDiva (BD) or Flowjo (Tree Star) software.

### GST pull down

To detect the interaction between ectopic expressed Bend5 and pluripotent factors (Oct4 and Sox2), HEK393T cells were transfected with HA-Bend5 and GST-Oct4 (or GST-Sox2). GST pull down was performed with GST beads (Glutathione Sepharose^TM^ 4B, 17-0756-01, GE healthcare), followed by Western blot with rabbit anti-HA and mouse anti-GST antibodies.

### Co-immunoprecipitation (Co-IP)

To detect Bend5 and Tet1 interaction, HEK293T cells were transfected with Flag-Bend5 and HA-Tet1. Immunoprecipitation was performed with rabbit anti-Tet1 antibody followed by Western blot with HA and Flag antibodies. To detect Bend4 and Oct4 interaction, HEK293T cells were transfected with Flag-Bend4 and GST-Oct4. Immunoprecipitation was performed with mouse anti-FLAG antibody followed by Western blot with rabbit Flag and mouse GST antibodies.

### GST tagged recombination protein purification and Electrophoretic gel-mobility shift (EMSA) assays

GST-Bend5 and GST-Bend5ΔBEN plasmid was transformed into *E*. *coli* strain BL21, and the fusion protein expression was induced by adding isopropyl thio-β-D-galactosidase (IPTG) in 1 mmol/L final concentration at 37 °C. After 2 h, cells were collected and the cell pellets were dissolved, followed by sonicator. Cells lysis were centrifuged at maximum speed, the supernatant and GST beads (Glutathione Sepharose^TM^ 4B, 17-0756-01, GE healthcare) incubation for 2 h at 4 °C, followed by wash, elution by specific buffer and SDS-PAFE detection. For EMSA, purified GST-tagged proteins (1 µg) were incubated with appropriate oligonucleotides (Table S3) (0.5 µg) in binding buffer (10 mmol/L Tris-Cl, pH 7.5, 50 mmol/L NaCl, 5 mmol/L MgCl_2_, 1 mmol/L DTT, 0.05% NP-40, 5% glycerol, 50 ng/μL poly (dI-dC)) for 20 min at room temperature. The reaction mixtures were then analyzed on agarose gels.

### Immunofluorescent staining

To detect the cell localization of Flag-Bend5 and Bend5∆BEN, the stably expressed cells were seeded on 15 mm microscope cover glasses. For immunofluorescent staining, cells were fixed with 4% paraformaldehyde, permeabilized (5% Triton-X, 20 mmol/L HEPES, 3 mmol/L MgCl_2_·6H_2_O, 300 mmol/L Sucrose), blocked (3% goat serum, 0.1% BSA in PBS) and incubated with rabbit anti-Flag antibody (GenScript A00170-40). Then they were stained with Alexa Fluor conjugated secondary antibodies Goat anti-Rabbit IgG (H + L). Nuclear staining was performed with DAPI.

### Reverse transcription and qPCR

Cells were dissolved by Trizol (Invitrogen) and total RNA was isolated using phenol chloroform. Following cDNA synthesis was performed with PrimeScript™ RT Reagent Kit (TaKaRa), and RT-qPCR was performed using GoTaq® qPCR Master Mix (Promega) and qPCR primers by ABI Step One Plus Real-Time PCR System. Gene expression data were normalized with Ct values from day 0 (D0) samples.

### Bisulfite sequencing and data analysis

For bisulfite sequencing, genomic DNA was extracted using the DNeasy Mini Kit (Qiagen) and processed using the EpiTect Bisulfite Kit (Qiagen) for bisulfite conversion. PCR amplification using specific primers, rTaq enzyme treatment, pEASYT1 cloning (TransGen Biotech) and sequencing were then performed as described previously (Lucifero et al., 2002). After bisulfite treatment, the gained DNA sequence was blasted with original sequence (unmethylated CG is changed to TG, methylated CG is not changed). We examined a total of 7 CpG sites within intron2 in *Igf2r* (GeneBank acc. no. L06446), 16 sites in the 5′ end of *H19* (GeneBank acc. no. U19619). Primer sequences are listed in the Table S3.

### Knocking out Bend4 in Bend5-expressing mESCs by CRISPR/Cas9

Bend5-expressing Stella-GFP mESCs were transduced with lentiviruses (pLenti-based vector) encoding Bend4 gRNA sequences targeting the BEN domain of Bend4 and selected with blasticidin. The px330-Cas9 plasmid (1 μg) (Addgene) was then electroporated into these cells to induce knockout. Cells were maintained in blasticidin for another 10 days. Single clones were subsequently isolated and verified for successful knockout by T7 endonuclease I (T7E1) assays and Sanger sequencing. *Bend4* gRNA: 5′-CCGCCGCCCGTCCTTCTTGG (AGG).

### RNA-seq and data analysis

The cells were harvested and dissolved in Trizol for total RNA extraction and treated with DNase I (Ambion) to remove any potential contaminated DNA fraction. The following library generation and sequencing were conducted by Ruibo Biotechnology Co., Ltd in Guangzhou. The raw data were used to map the RNA-seq reads to the UCSC mouse genes (mm10). Cuffdiff (version 2.0.2) was employed to calculate expression abundance measured as Fragment Per Kilobase per Million mapped fragments (FPKM) and identify the DE genes (fold change > 2) (Table S4). The consistency degree of gene expression profiles was measured by Pearson correlation coefficient and visualized by scatter plots. Pearson correlation analysis was performed based on gene expression levels (FPKM) and visualized by heatmaps.

### Chromatin immunoprecipitation followed by sequencing (ChIP-seq) and data analysis

ChIP-seq was performed as previously described (Yao et al., 2018). Briefly, the cells (2 × 10^7^) were collected, then crosslinked by 1% Formaldehyde solution (Sigma-Aldrich F8775) for 10 min and quenched by final concentration 250 mmol/L glycine solution. The cells were lysed in ChIP lysis buffer (50 mmol/L Tris pH 8.0, 10 mmol/L EDTA, 1%SDS, protein inhibitor cocktail) and sonicated (Bioruptor for 30 cycles of on (30 s)/off (30 s)), then crosslinking. Cell lysis was dialyzed in ChIP dilution buffer (0.01% SDS, 1.1% Triton X-100, 1.2 mmol/L EDTA, 16.7 mmol/L Tris-HCl pH 8.0, 167 mmol/L NaCl) at 4 °C for 4 h before incubation with the appropriate antibodies. Each reaction was sequentially washed with low salt buffer (0.1% SDS, 1% Triton X-100, 2 mmol/L EDTA, 20 mmol/L Tris-HCl pH 8.0, 150 mmol/L NaCl ), high salt buffer (0.1% SDS, 1% Triton X-100, 2 mmol/L EDTA, 20 mmol/L Tris-HCl pH 8.0, 500 mmol/L NaCl), LiCl buffer (0.25 mol/L LiCl, 1% Igepal, 1 mmol/L EDTA, 10 mmol/L Tris-HCl pH 8.0, 1% deochyacid) and TE buffer (10 mmol/L Tris-HCl pH 8.0, 1 mmol/L EDTA) at 4 °C. Co-precipitated DNA was recovered using Mini-Elute PCR Purification Kit (28004, Qiagen). About 10 ng IPed DNA and input DNA measured by Qubit Fluorometer (Invitrogen) were used to construct DNA library. The following library generation and sequencing were conducted by Ruibo Biotechnology Co., Ltd in Guangzhou.

In this study, anti-Flag, Tet1, and CTCF ChIP-seq experiments were performed. Oct4 ChIP-seq data (GSE56098, GSM1355154, GSM1355155, GSM1355167) were download from the NCBI website. All raw data were uploaded to Galaxy server (http://usegalaxy.org) to perform reads quality check by the FastQC software integrated into the tool shed of the Galaxy server home page (Afgan et al., 2018). Low-quality reads and Illumina universal adapters were removed by Trim Galore. Then control input and ChIPed clean data were respectively mapped by Bowtie2 to the built-in index of mm10 mouse reference genome. The Bam files obtained from the Bowtie2 software were filtered by the Bam/Sam filter software to remove low-quality data (<20) and those mapped to the mitochondrial genome. To better visualize the results, the filtered Bam files were converted to the bigWig file format with bin size 5 by the bamCoverage software and displayed in the Integrative Genomic Browser (IGB). All tracks were selected and synchronized for the same Y-axis value (no expression) which demonstrated the lower and upper range values for any base position in the current view. The results of transcript factor binding events was summarized by the MACS2 software. The result files with bed format were obtained from MACS2 on Bam files with the threshold of FDR > 0.05 (False Discovery Rate). The last annotation step was performed in the Linux system by the Homer software and gene ontology analysis was conducted through the MEME website.

### Assay for transposase-accessible chromatin using sequencing (ATAC-seq) and data analysis

ATAC-seq was performed as previously described (Buenrostro et al., 2013). *Stella-GFP* positive EpiLCs were sorted after EpiLC induction D2, about 1 × 10^4^ cells were collected and centrifugation at 500 ×*g* for 5 min, which was followed by a wash using cold PBS and centrifugation at 500 ×*g* for 5 min. The cells were lysed using 50 µL of cold lysis buffer (10 mmol/L Tris-HCl, pH 7.4, 10 mmol/L NaCl, 3 mmol/L MgCl_2_ and 0.2% IGEPAL CA-630) for 10 min on ice, then nuclei were collected at 500 ×*g* for 10 min. The pellet was immediately resuspended in the transposase reaction mix (Vazyme, TD502 kit, 20 µL each reaction), then the reaction was carried out for 30 min at 37 °C. The sample was purified using 40 µL magnetic beads (Beckman Coulter AMPure XP A63881). Following purification, we amplified library fragments using Vazyme, TD502 kit (50 µL each reaction), using the following PCR conditions: 72 °C for 3 min; 98 °C for 30 s; and thermocycling at 98 °C for 15 s, 60 °C for 30 s and 72 °C for 30 s for 15 cycles, then 72 °C for 5 min. Following PCR, the libraries were purified using 60 µL magnetic beads. The purified libraries were detected by agarose gel electrophoresis and final concentration was detected by Qubit 3.0 (Invitrogen). The sequenced insert size was between 40 bp and 1 kb with a mean of ~120 bp. Following library QC and quantification, sequencing was performed by Guangzhou RiboBio Co., Ltd.

The reads were aligned to mm10 using Bowtie2 and only unique aligned reads were collected. For all data, duplicates were removed using Picard. Then mitochondrial sequences were removed and peaks were called using dfilter (Kumar et al., 2013). BigWig files were produced using genomeCoverageBed and displayed in the Integrative Genomic Browser (IGB).

## Supplementary Information

Below is the link to the electronic supplementary material.

Supplementary file1 (PDF 1555 kb)

Supplementary file2 (XLSX 827 kb)

Supplementary file3 (XLSX 12 kb)

Supplementary file4 (XLS 28 kb)

Supplementary file5 (XLSX 2171 kb)
