# Supplementary material for: Bend family proteins mark chromatin boundaries and synergistically promote early germ cell differentiation
Source: Protein Cell. 2021 Nov 3;13(10):721–41. doi: 10.1007/s13238-021-00884-1 (PMC9233729; doi:10.1007/s13238-021-00884-1)

Supplementary Figure Titles and Legends

Fig. S1. A coupled genome-wide screening strategy using BiFC and germ cell differentiation assays, Related to Fig. 1.

(A) For each bait, its interaction with every ORF was converted to a weighted positive ratio (WPR) (y-axis) using CytoArray. A cutoff value (at 90% confidence level) was then selected for each bait (indicated by the horizontal red line). ORFs that scored above the cutoffs (with their assigned numbers on the x-axis) were plotted with blue peaks to indicate their WPR values. Arrows indicate the positions of select genes identified from the screens.

(B-C) *Stella*-GFP mESCs stably expressing human NME7, C4ORF27, HOXA3, and BEND5 were cultured in EpiLC induction medium and either analyzed at day 2 by FACS (B), or cultured for an additional day in PGCLC-inducting media before analysis by microscopy (C). Error bars represent mean±SD, n=3 independent experiments. $^{**}P < 0.01$ (two-tailed *t* test).

Fig. S2. Bend5 promotes EpiLCs induction and *Stella* activation, Related to Fig. 2.

(A) The relative expression level of Bend5 in MEFs, mESCs, and PGCs (left) as well as mouse tissues (right) was measured by RT-qPCR. Error bars represent mean±SD; n=3 independent experiments. $^{**}P < 0.01$, $^{***}P < 0.001$ (two-tailed *t* test).

(B) YFPN or YFPC-tagged candidate genes were stably co-expressed in HTC75 cells as indicated. Scatter plots from flow cytometry analysis are shown here. Sox2/Oct4 co-expression was used as positive control.

(C-D) _Stella-GFP_ reporter mESCs stably expressing vector control or Flag-tagged _mouse_ Bend5 were differentiated into EpiLCs. GFP-positive cells were sorted at day 0 and 2 for total RNA extraction and relative gene expression analysis by RT-qPCR for the indicated genes. Error bars represent mean±SD; n=3 independent experiments. **_P_ <0.01; ***_P_ <0.001 (two-tailed _t_ test).

(E) Embryoid body (EB) formation was performed for 7 days using mESCs stably expressing vector control or Flag-Bend5. The relative expression of Bend5 was detected by real-time qPCR (left). Stella-GFP (SG+)-positive cells were quantitated by flow cytometry at day 7 (right). Error bars represent mean±SD; n=3 independent experiments. **_P_ <0.01, ***_P_ <0.001 (two-tailed _t_ test).

(F) _Blimp1_-Vensus (BV) and _Stella-CFP_ (SC) dual-reporter mESCs stably expressing vector control or Flag-Bend5 were induced to differentiate into EpiLCs and embryoid bodies. BVSC double positive cells at day 2 after EpiLC induction (left) or at day 7 of EB formation (right) were analyzed by flow cytometry. Error bars represent mean±SD; n=3 independent experiments. *_P_ <0.05 (two-tailed _t_ test).

(G) _Bend5_ was knocked down _Blim_p_1_-Vensus and _Stella-CFP_ dual-reporter mESCs and the cells were induced to differentiate into EpiLCs. _Bend5_ mRNA expression was detected by RT-qPCR (left) and _BVSC_ double positive cells were quantitated by flow cytometry. Error bars represent mean±SD ; n=3 independent experiments. *_P_ <0.05, **_P_ <0.01 (two-tailed _t_ test).

(H) The relative mRNA expression level of _Fos_, _Fosb_, and _Fgf5_ in mouse MEFs, ESCs, and PGCs was measured by RT-qPCR. Error bars represent mean ± SD; n=3

independent experiments. ***P <0.001 (two-tailed t test).

Fig. S3. Bend5 BEN domain deletion decreases *Stella* activation, Related to Fig. 3 and Fig. 4.

(A-B) S/L mESCs and 2i/L mESCs at different passages were collected for genomic DNA extraction. The bisulfite sequencing was performed to assess the CpG methylation level of the H19 and Igf2r loci.

(C) Bend5 overexpressed cells or control 2i/L mESCs at P1 were performed to EpiLC induction, then the cells were collected for genomic DNA extraction. The bisulfite sequencing was performed to assess the CpG methylation level of the H19 and Igf2r loci.

(D) Relative Tet1 mRNA expression was determined in control and Flag-Bend5-expressing cells before and after EpiLC induction.

(E) mESCs stably expressing vector control or Flag-Bend5 were cultured for EpiLC induction. Cells were collected for anti-Tet2 ChIP-qPCR assays at the indicated time points to assess Tet2 occupancy on the *Stella* locus. Error bars represent mean±SD; n=3 independent experiments.

(F) *Stella*-GFP reporter mESCs stably expressing Flag-Bend5, Flag-Bend5ΔBEN, or empty vector were immunostained with an anti-Flag antibody. DAPI was used to stain the nuclei.

(G) *Stella*-GFP reporter mESCs stably expressing Flag-Bend5, Flag-Bend5ΔBEN, or empty vector were cultured for EpiLC induction for two days. Bright-field and

fluorescence imaging was done at day 0 and 2.

(H) Bacterially purified GST-tagged Bend5 and Bend5ΔBEN were resolved by SDS-PAGE and stained with Coomassie blue. About 2 μg protein was loaded into each lane. GST alone served as negative control. The proteins were then used for electrophoretic gel-mobility shift assay.

(I) Bacterially purified GST-tagged full-length Bend5 or Bend5ΔBEN proteins (~1 μg) were incubated with various oligos (0.5 μg) and the reaction mixtures were resolved on agarose gels. Oligos 1-4 (~30bp) were based on genomic sequences within primer set 2 (Fig. 4A). Oligo 5 (~30bp) corresponds to the *Stella* TSS region. Bracket indicates shifted oligos.

Fig. S4. *Bend4* is highly expressed in PGC and mESCs, Related to Fig. 5.

(A) The relative expression level of BEN family genes in MEFs, mESCs, and PGCs was measured by RT-qPCR. Error bars represent mean ± SD ; n=3 independent experiments. **P* <0.05, ***P* <0.01 (two-tailed *t* test).

(B) The relative expression level of *Bend4* in mouse tissues was measured by RT-qPCR. Error bars represent mean±SD ; n=3 independent experiments. ***P* <0.01, ****P* <0.001 (two-tailed *t* test).

(C) *Bend4* isforms mRNA were detected in testis and mESCs by RT-PCR. The arrows indicated different *Bend4* isforms. The *Gapdh* were shown as internal control.

(D) Bend5 and Bend4 were individually and stably expressed in *Stella*-GFP reporter mESCs and detected by western blotting with anti-Flag and Gapdh antibodies.

(E) Bend5 and Bend4 were co-expressed in *Stella*-GFP reporter mESCs and detected by western blotting with anti-Flag and Gapdh antibodies.

(F) To disrupt Bend4 function, a guide RNA was designed to target the exon encoding the BEN domain. Because of the lack of a good antibody against endogenous Bend4, the isolated clones were sequence verified. Listed are the genomic sequences of their respective target regions. Brackets indicate the regions deleted in the clones.

Fig. S5. Bend4 and Bend5 bind to PGC related genes, Related to Fig. 6.

(A-B) mESCs stably expressing Flag-Bend4 or Bend5 were cultured for EpiLC induction and collected at day 0 and 2 for ChIP-seq using an anti-Flag antibody. The binding sites of Bend4 and Bend5 on the *Tfap2c* (A) and *Prdm14* (B) gene loci are shown. Red arrows indicate Bend4/Bend5 peaks.

(C) CentriMo Motif analysis of Bend4 and Bend5 binding motifs compared to Insv.

(D) Peak overlapping analysis of Bend4 and Bend5 peaks in the genome was performed. The ratio (%) of overlapping peaks (Y-axis) was calculated and shown.

(E) Sequence alignment of Bend4 and Bend5 BEN domains.

(F) Consensus binding motifs of Bend4 and Bend5 at day 0 and 2 were aligned on *Stella* locus.

Fig.S6. Bend5 and Bend4 are a new family of chromatin boundary factors, Related to Fig. 7.

(A) ATAC-seq was performed using Flag-Bend4 or Bend5-expressing mESCs cultured

in EpiLC inducing media. Flag ChIP-seq and ATAC-seq peak overlapping analysis was performed in various genomic regions. The ratio (%) of overlapping peaks (Y-axis) was calculated and shown.

(B) Overlapping of Flag ChIP-seq and ATAC-seq peaks was analyzed for PGC-related gene loci. Green, ATAC-seq peaks. Blue, Flag ChIP-seq peaks.

(C) Overlapping peaks between Flag and CTCF ChIP-seq were analyzed at PGC-related genes loci (*Prdm14*, *Lin28a*, *Tfap2c*). Red, CTCF ChIP-seq peaks. Blue, Flag ChIP-seq peaks.

Supplementary Fig. S1

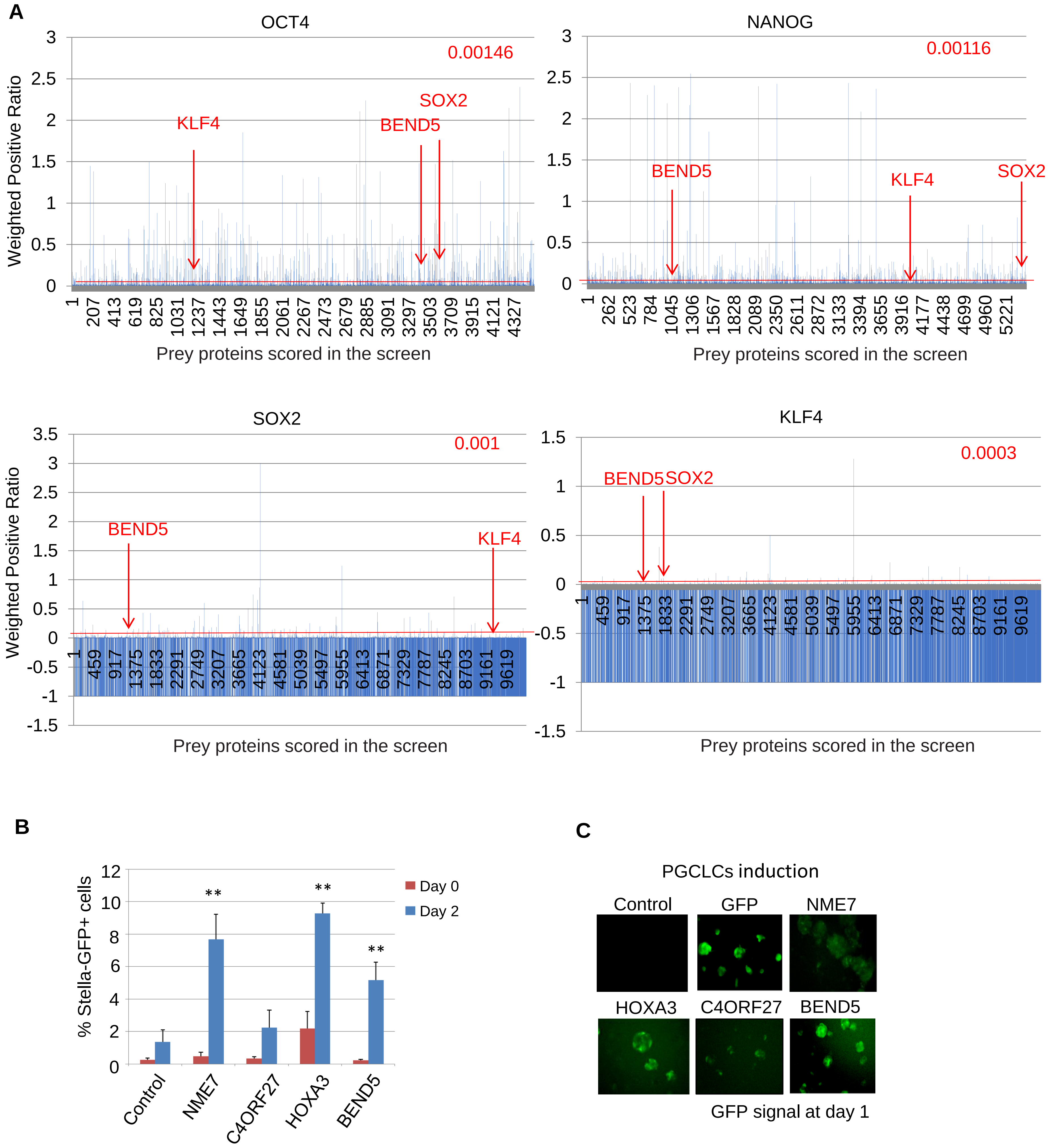

Supplementary Fig. S2

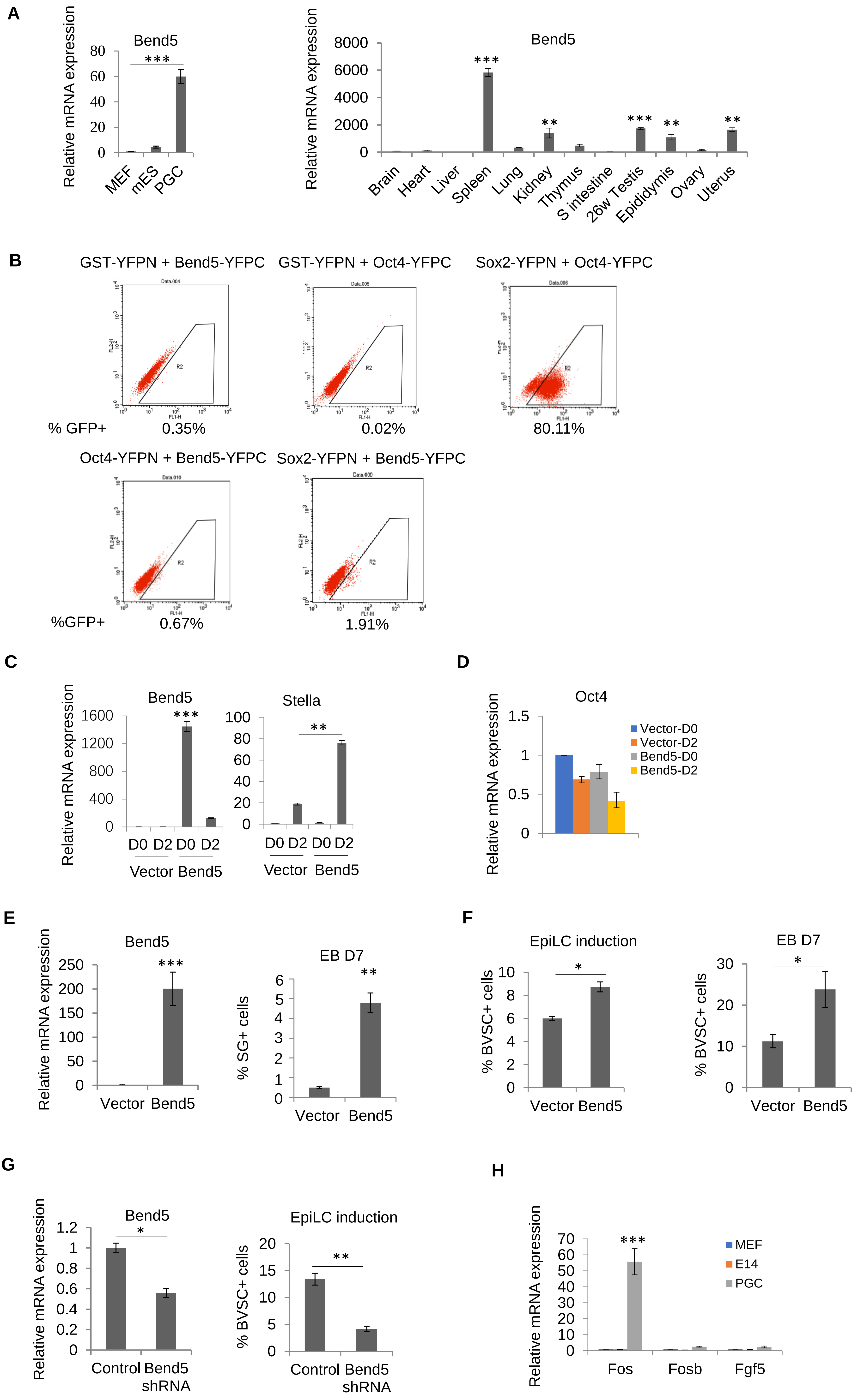

Supplementary Fig. S3

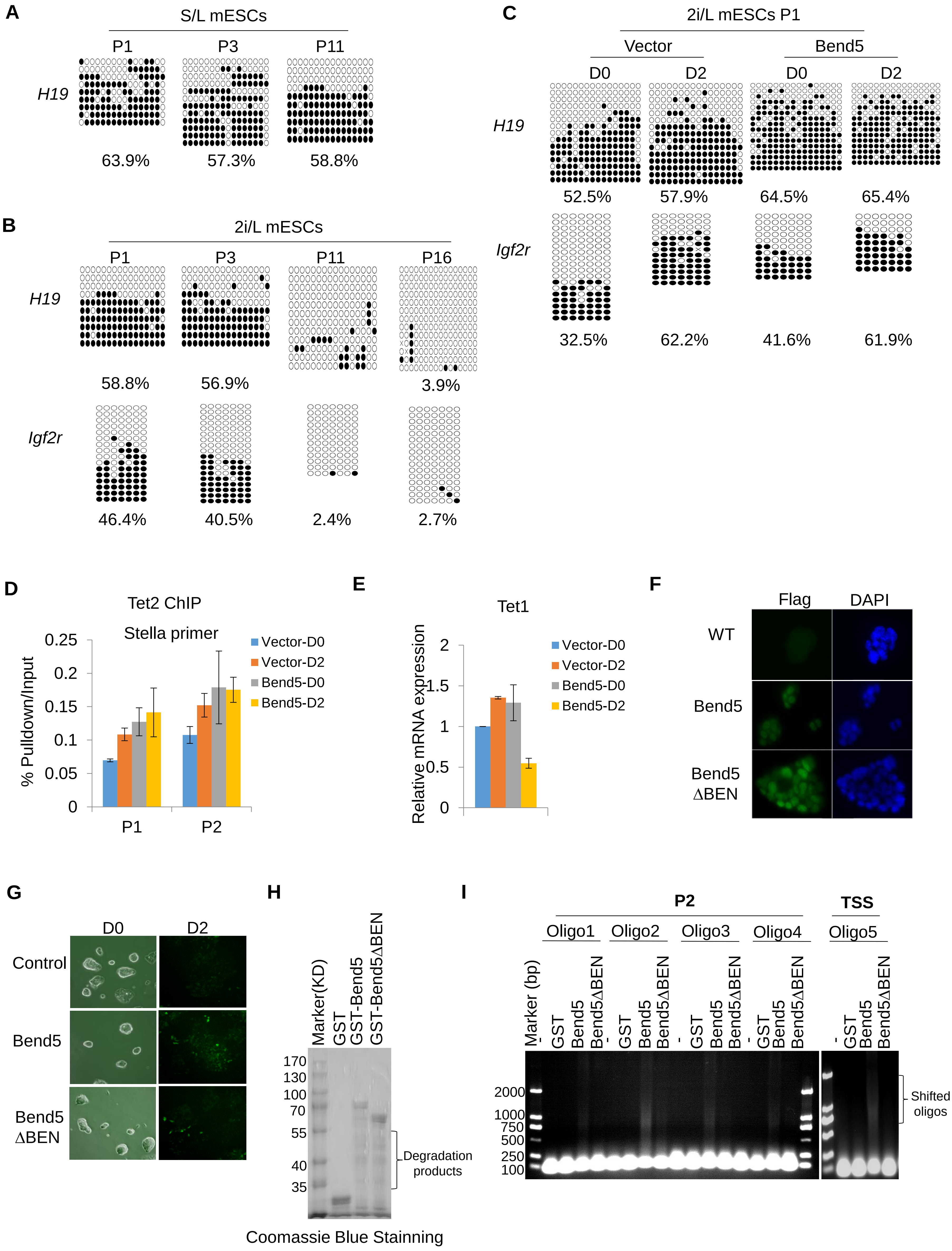

Supplementary Fig. S4

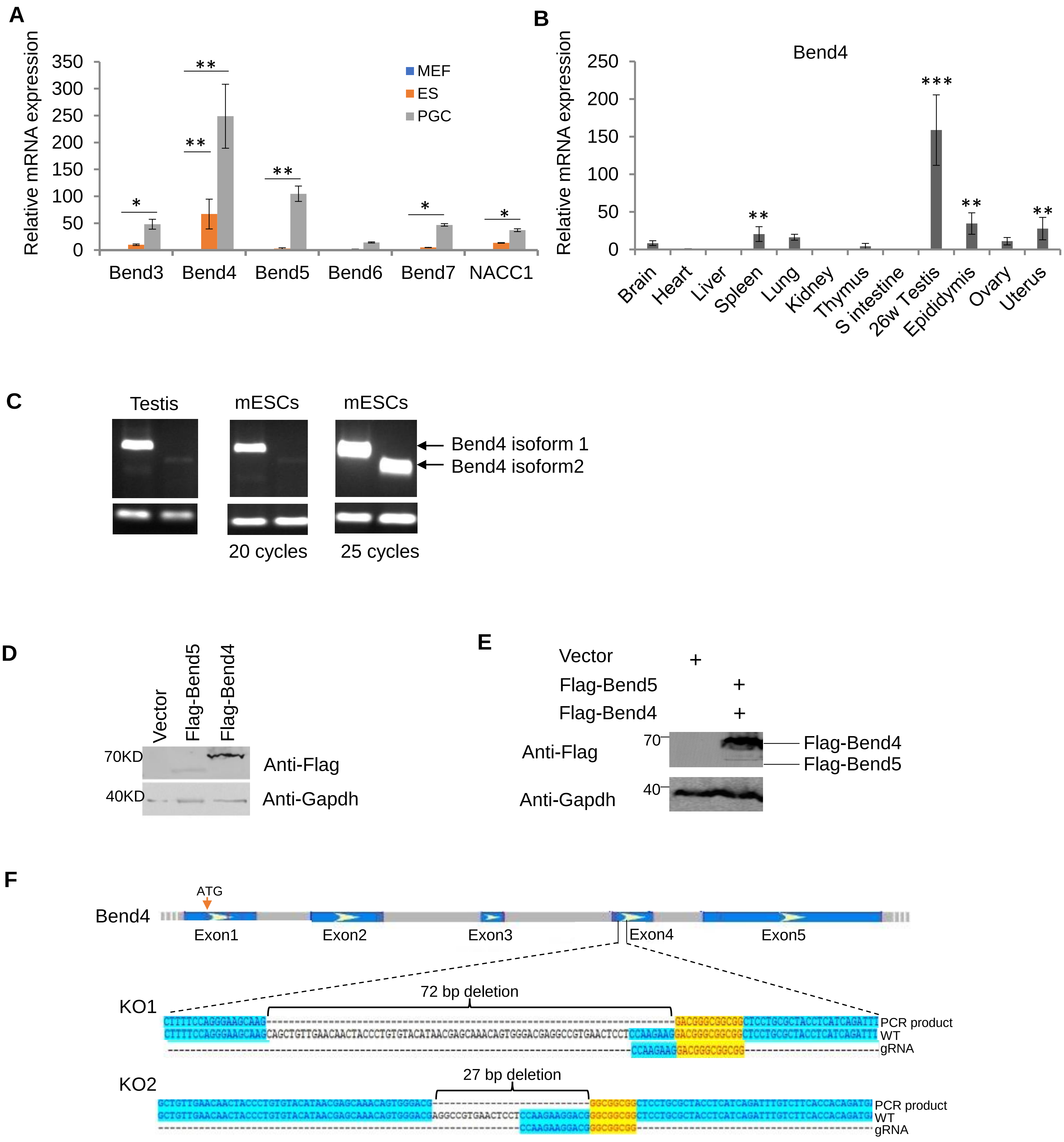

Supplementary Fig. S5

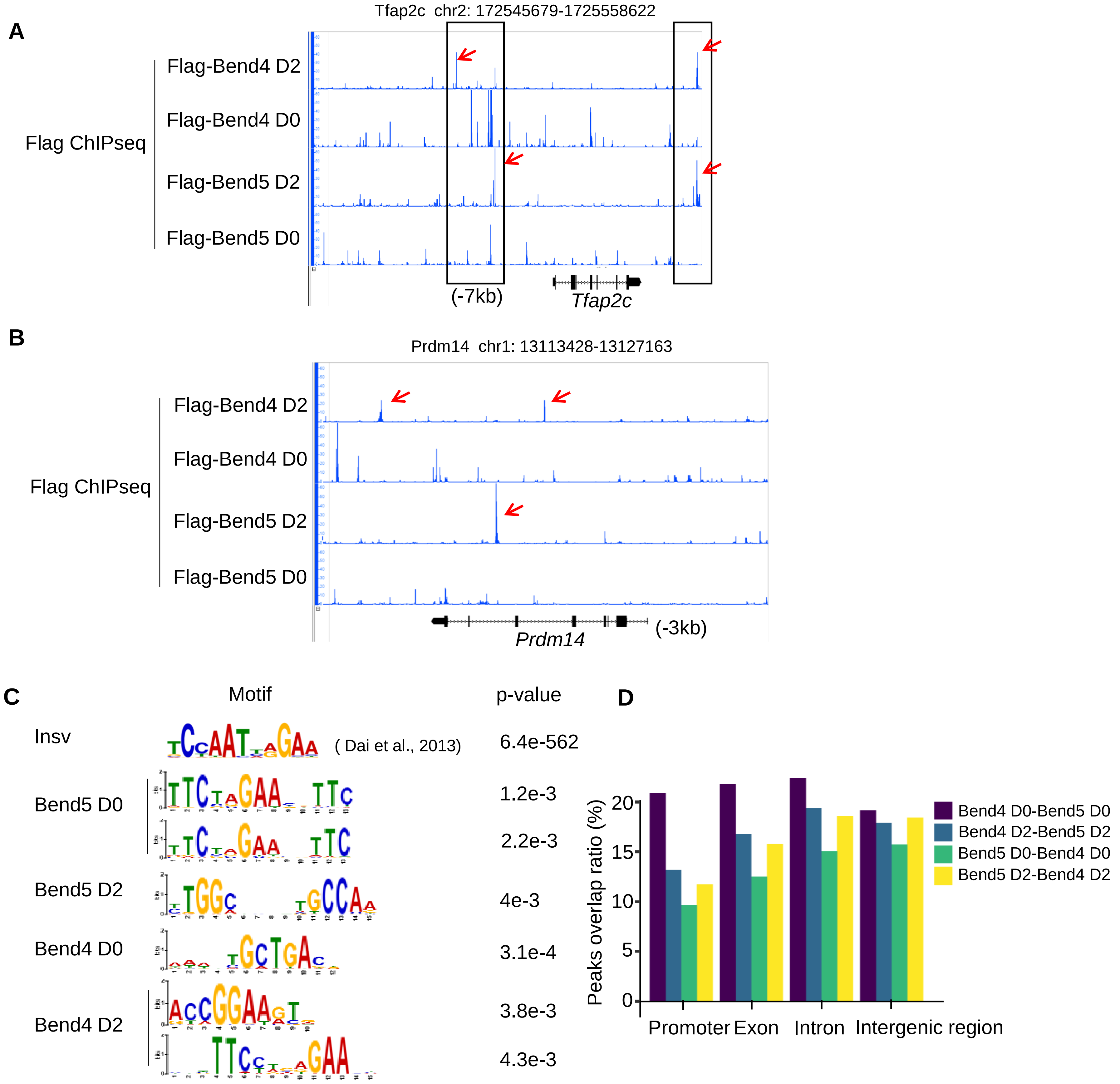

E

Bend4
>sp|P86174|398-506-mouse-Bend4 BEN domain
NYPVYITSKQWDEAVNSSKKDGRRLLRYLIRFVFTTDELKYSCGLGKRKRSVQSGE
TGPERRPLDPVKVTCLREFIRMHCTSNPDWWMPSEEQINKVFSDAVGHARQGR
Bend5
>sp|Q8C6D4|302-408-mouse-Bend5 BEN domain
GSGIWVDEEKWHQLQVTQGDSKYTKNLAVMIWGTDVLKNRSVTGVATKKKKDAIPK
PPLSPHKLSIVRECLYDRIAQETVDETEIAQRLSKVNKYICEKIMDINKSC

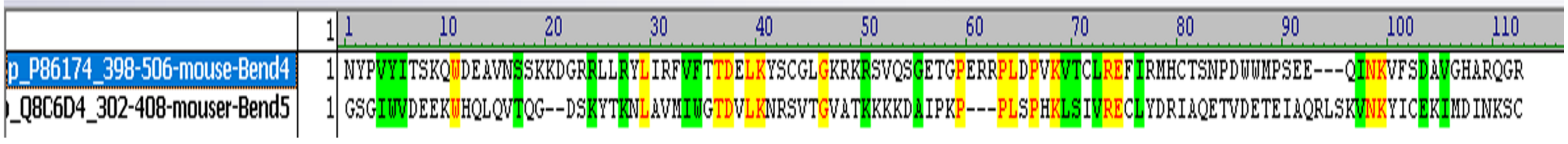

F

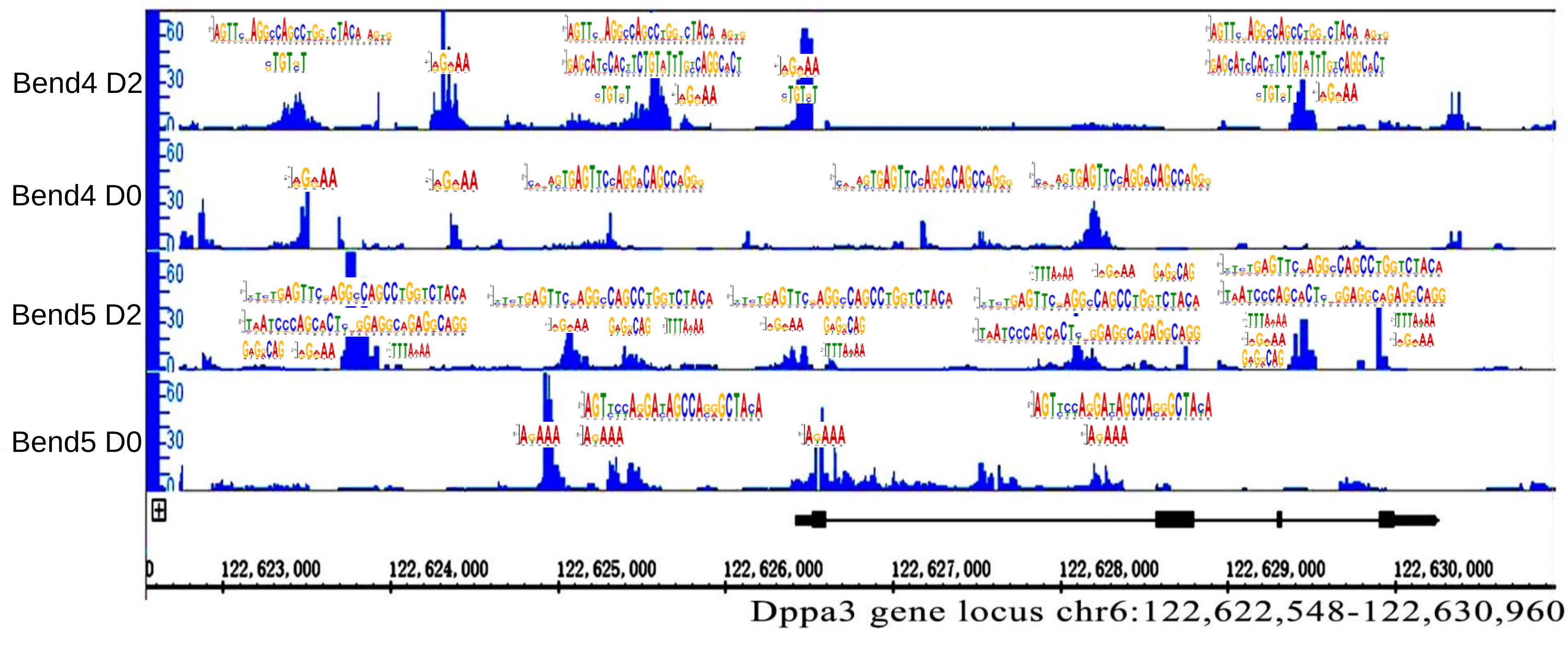

Supplementary Fig. S6

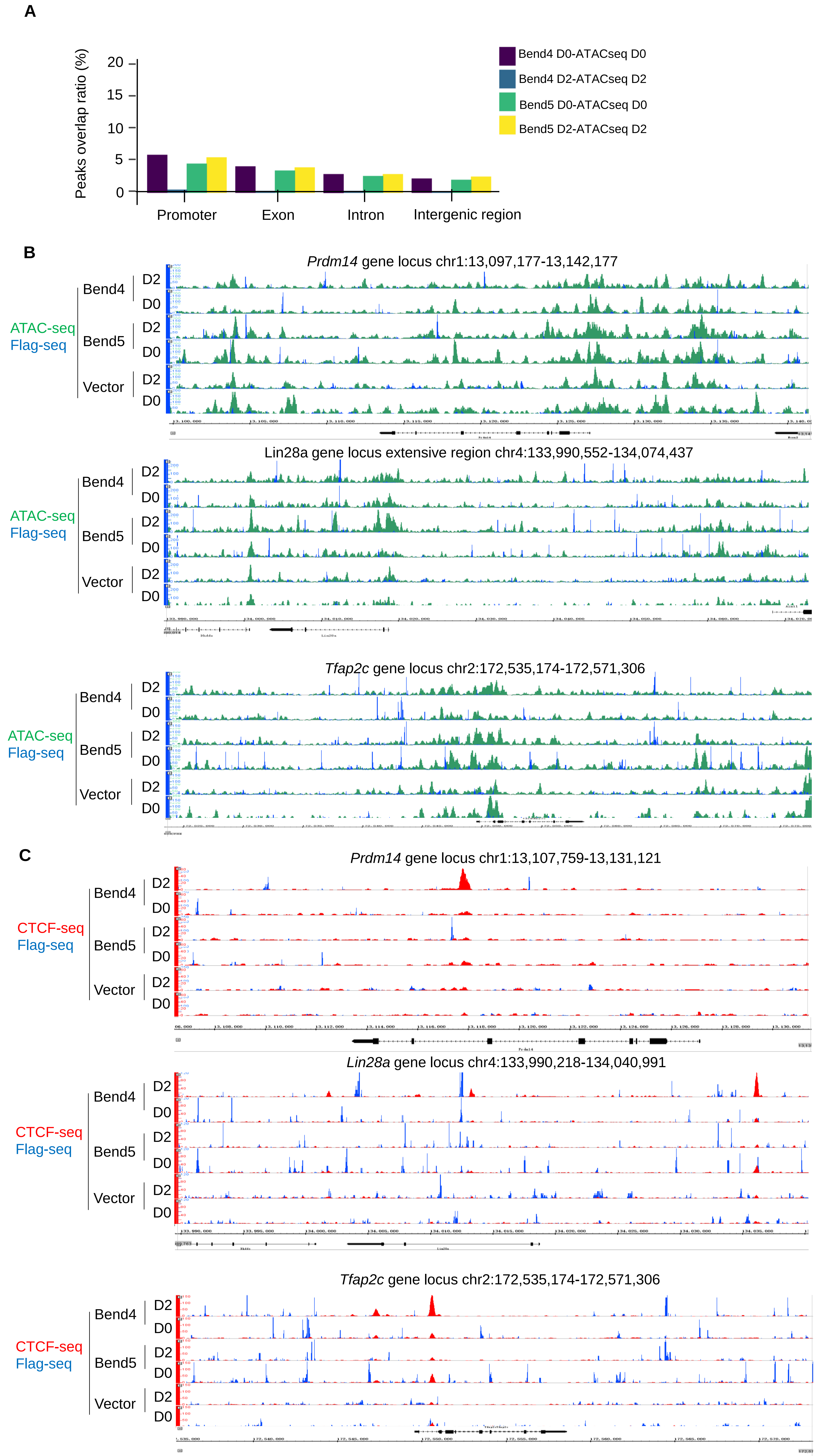

Supplement: Supplementary file 1 — Supplementary file1 (PDF 1555 kb) [file 13238_2021_884_MOESM1_ESM.pdf]
